# Effects of *Chardonnay* grape skin residue on the chemical, sensory and nutritional value of *Cabernet Sauvignon* wine^☆^

**DOI:** 10.1016/j.fochx.2025.103043

**Published:** 2025-09-19

**Authors:** Xiaochun Zheng, Jie Sheng, Yu Chen, Bin Wang, Xuewei Shi

**Affiliations:** a College of Enology, Northwest A&F University, Yangling 712100, Shaanxi, PR China.; b Food College, Shihezi University, Shihezi, 832000 Xinjiang, Uygur Autonomous Region, PR China.; c College of Food Science and Technology, Huazhong Agricultural University, Wuhan, Hubei 430070, PR China.

**Keywords:** *Cabernet sauvignon* wine, *Chardonnay* grape skin residue, Chemical sensory properties, Nutritional characteristics, *In vitro* digestion, Bioaccessibility

## Abstract

This study explored the impact of adding *Chardonnay* grape skin residue (CGSR) on the quality of *Cabernet Sauvignon* wine. The results showed that the physicochemical indexes, aroma substances, and taste of *Cabernet Sauvignon* wine with CGSR (CSWS) were significantly improved. The content of volatile organic compounds (VOCs) in CSWS increased by 34.1 %. The total antioxidant capacity of CSWS increased by 17.7 %. *In vitro* simulated digestion experiments indicated that the bioaccessibility of CSWS increased by 7.5–9.2 %. Based on 50 shared differential metabolites in non-targeted metabolomics, changes in the contents of amino acids, flavonoids, and organic acids reflected the specific characteristics of the wine. This study shows that adding CGSR can not only improve the flavor quality of *Cabernet Sauvignon* wine but also increase the value of CGSR, and provide a new strategy for enhancing the complexity of red wine and exploring its nutritional characteristics.

## Highlights

• *Chardonnay* grape skin residue affected the quality of *Cabernet Sauvignon* wine.
• CSWS produced more aroma substances and improved the flavor.
• Some of the amino acids and flavonoids were enhanced in CSWS.
• The metabolic pathway of CSWS was analyzed using metabolomics.
• Arginine and proline, aspartate metabolisms were enhanced after *in vitro* digestion.

## 1 Introduction

The global wine industry is increasingly prioritizing sustainability, driving innovations to valorize winemaking byproducts while enhancing product quality. Grape pomace, comprising skins, seeds, and stems, constitutes 20–30 % of processed grapes and is traditionally discarded or underutilized despite its rich reservoir of bioactive compounds, including phenolics, polysaccharides, and aroma precursors (Lopes et al., 2025). The global wine industry generates approximately 8–10 million tons of grape pomace annually, with grape skin residues accounting for 60–70 % of this byproduct (Constantin et al., 2024). While traditional disposal methods (*e.g.*, composting, animal feed) dominate current practices, emerging research highlights the untapped potential of grape skins as value-added ingredients in winemaking. Particularly, the reintegration of grape skin residues into fermentation processes has shown promise in modulating wine composition through polyphenol leaching, microbial activity regulation, and structural modification of tannin complexes (Ilyas et al., 2021; Sancho-Galán et al., 2021).

*Cabernet Sauvignon,* a cornerstone of premium red wine production, is prized for its bold tannic structure and dark fruit aromas (Tu et al., 2022; Zhao et al., 2023). However, in cooler climates or under suboptimal ripening conditions, *Cabernet Sauvignon* wines may exhibit excessive astringency or herbaceous notes, limiting their sensory appeal (Barnuud et al., 2014; Costa et al., 2020). Generally speaking, the addition of red grape skin residues often leads to an imbalance in the tannin content of red wine (Tu et al., 2025). In contrast, white grape skin residue, with lower tannin levels but higher glycosylated aroma potential, offers a safer route for aroma enhancement without compromising tannin balance (Mosele et al., 2023; Polat et al., 2022; Radulescu et al., 2024). *Chardonnay* grape skin residue (CGSR), a byproduct of white winemaking, is rich in hydroxycinnamic acids (*e.g.*, caftaric acid), flavonols (*e.g.*, quercetin glycosides), and glycosidically bound volatile compounds (*e.g.*, terpenes (*e.g.*, linalool, geraniol), norisoprenoids (*e.g.*, β-damascenone)) that remain largely intact post-pressing (Buican et al., 2025; Liang et al., 2020; Zemni et al., 2024). These bound volatiles can be enzymatically hydrolyzed during fermentation, releasing free aromatic compounds that enhance floral and fruity nuances. Furthermore, the relatively high levels of fruit acids in CGSR can stabilize wine color *via* copigmentation while mitigating astringency through polysaccharide-tannin interactions (Vendramin et al., 2021). Compared to red varieties, high concentrations of hydroxycinnamic acids, flavonols, and oligosaccharides exhibit demonstrated capacities to influence fermentation kinetics through yeast nutrient supplementation, modulate color stability *via* copigmentation effects, and enhance antioxidant capacity through synergistic interactions with native *Cabernet* phenolics (Gençdağ et al., 2022; Holt et al., 2022; Yue et al., 2021).

Therefore, the addition of CGSR to *Cabernet Sauvignon* could introduce multiple functional components: (1) phenolic compounds such as hydroxycinnamic acids and flavonols, which modulate tannin polymerization and color stability (Li et al., 2021); (2) polysaccharides and caftaric acid that interact with tannins to reduce astringency (Kuhlman et al., 2024). Meanwhile, these compounds hold multiple potentials: (1) phenolic interactions with *Cabernet Sauvignon* tannins to refine mouthfeel; (2) enzymatic or acidic hydrolysis of bound aromas during fermentation to enhance volatile diversity; and (3) enrichment of nutritional antioxidants. Integrating CGSR into *Cabernet Sauvignon* fermentation thus represents a multifaceted opportunity to align waste valorization with sensory and nutritional enhancement—an approach yet to be systematically explored. Nevertheless, the strategic incorporation of *Chardonnay* skins into *Cabernet Sauvignon* vinification remains unexplored, particularly regarding its dual impacts on sensorial attributes and nutritional enhancement.

To uncover how adding CGSR influences the quality of *Cabernet Sauvignon* wine, this study sets out with a clear focus: first, measuring the wine's basic physicochemical properties, volatile organic compounds (VOCs), and organic acids lays the groundwork to understand how CGSR shapes its character; secondly, electronic sensory detection was employed to analyze changes in aroma and taste, and non-targeted metabolomics was used to study changes in metabolites; next, we explore—for the first time—how adding CGSR affects the bioaccessibility and digestion of the wine's metabolites under simulated *in vitro* conditions, with an eye to their potential health impacts. This study not only delivers a sustainable, actionable pathway for the recycling of CGSR but also presents a novel strategy to boost the complexity of *Cabernet Sauvignon* red wine.

## 2 Materials and methods

### 2.1 Grape collection and winemaking

The grapes utilized, including *Cabernet Sauvignon* (CS) and *Chardonnay* (CH) varieties, were collected from the vineyard of CITIC Guoan Wine Co., Ltd. (Manas County, Changji Hui Autonomous Prefecture, Xinjiang Uygur Autonomous Region, China) (E86.21°, N44.30°). The vineyard experiences an average annual temperature of 7.2 °C and an average annual precipitation of 173.30 mm, with the precipitation mainly concentrated from July to September. Grape berries were harvested once they reached optimal technological maturity, which was determined by the sugar-to-acid ratio concentrations. In August 2023, approximately 100 kg of grape berries for each variety were obtained and promptly transported to the laboratory for further winemaking. Each variety comprised three replicate samples. Each sample, weighing around 30 kg, was randomly gathered from fifteen grapevines (three grapevines at each of the five sampling points) following the five-point sampling approach. Evenly spread CH grapes in a dry, clean, perforated plastic basket (1 m × 0.5 m × 0.5 m). Place it on a 2 m high shelf in a well-ventilated room with 50–55 % relative humidity for natural air-drying until the weight is constant.

In this study, the effect of adding CH grape skin residue on CS wine production was investigated. As a result, two types of wines were produced: CS wine (CSW) and CSW with CH grape skin residue (CSWS). The winemaking process of CSW was carried out using a modified laboratory-based method as reported by the previous study (Chen et al., 2024). Briefly, each 30 kg of grape berries were destemmed, crushed and transferred into a 35-L sterilized tank (Xiangsheng plastic products Co., LTD, Yutian, Hebei, China), in triplicate. The musts were treated with 60 mg/L sulfur dioxide and 100 mg/L of pectinase (Lallzyme EX-V, Lallemand, France) to clarify the grape juice at 8 °C. After 24 h of maceration, 200 mg/L activated commercial yeast (1 × 10^7^ cells/mL, *Saccharomyces cerevisiae* L2226, Angel Yeast Co., Ltd., China) was inoculated into the grape must without pomace to start alcohol fermentation at 25–28 °C. Unlike CSW, CSWS contained an additional 10 % (3.5 kg) of dried CH grape skin residue. Once the residual sugar was showed no fluctuations for two consecutive days, the wine was transferred to sterile tanks, treated again with 50 mg/L sulfur dioxide, clarified, stored at 4 °C for 3 months, and then analyzed.

### 2.2 Conventional chemical profiles

Titratable acidity (TA), Soluble solids (SS), and pH values were measured according to the previous method without any modification (Chen et al., 2022). High-performance liquid chromatography (HPLC) (An LC-2050C 3D instrument (Shimadzu, Japan) equipped with an Aminex HPX-87H column) was employed to conduct quantitative analysis of residual sugar (RS, the total amount of glucose and fructose), ethanol, glycerol, and organic acids (including tartaric acid, succinic acid, malic acid, and lactic acid) (Chen, Lei, Sun, et al., 2025). The mobile phase was 5 mmol/L H₂SO₄, flowing at 0.6 mL/min. The column was set at 60 °C for a 35 min run. For each 10 μL sample injection, signals were detected by a Shimadzu VWD UV detector (PDA for organic acids; 210 nm) and an RID-20 A refractive index detector (RID for hexoses and alcohols). The contents of total phenolics (TPH), total flavanols (TFA), total flavonols (TFO), and total tartrate esters (TTA) in wines were measured by previously reported methods for phenolic parameter analysis (Sheng et al., 2024). All analytical reagents and chromatographic standards were purchased from Sigma-Aldrich (St. Louis, Missouri, USA), with a purity >98 %. The water for all analyses was commercially available purified water from Hangzhou Wahaha Group Co., Ltd., China.

### 2.3 Determination of aroma compounds

Aroma composition of wine samples was identified and quantified by solid phase microextraction-gas chromatography–mass spectrometry (SPME-GC–MS) (Thermo Fisher Scientific, USA) method with the previous study (Chen, Lei, Zhang, et al., 2025). Briefly, 5 mL samples were mixed with 1.20 g NaCl and 10 μL of 4-methyl-2-pentanol (1.03 g/L) in a vial. Following equilibration at 40 °C for 30 min, the autosampler was set to SPME mode. A DVB/CAR/PDMS fiber (50/30 μm; Thermo Fisher Scientific, USA) was used to extract volatile compounds with shaking at 250 rpm for 30 min. Compounds were separated using a TG-WAX capillary column (60 m × 0.32 mm × 0.25 μm; Thermo Fisher Scientific, USA). Chromatographic separation was conducted with the following program: desorption for 8 min, initial column temperature held at 50 °C for 5 min, followed by ramping to 230 °C at 8 °C/min, and final temperature maintained for 10 min. MS analysis used positive electron ionization (EI) mode (29–350 *m*/*z*) with helium as the carrier gas at a flow rate of 1.0 mL/min. Temperatures for the ion source, quadrupole, and mass selective detector (MSD) transfer line were maintained at 230 °C, 150 °C, and 250 °C, respectively. The key volatile compounds in the wine were identified and matched by comparing their retention indices with those of standard compounds (purchased from Sigma-Aldrich, USA) and the NIST17.0 library. Their concentrations were quantified using calibration curves.

### 2.4 Analysis of electronic senses

Electronic sensing tests were conducted with minor modifications to the method of the previous study (Chen et al., 2023). The electronic tongue (ASTREE II, Alpha-MOS, France) was equipped with seven chemical sensors, namely AHS for detecting sourness, CPS for “Synthesize 1”, NMS for umami, CTS for saltiness, PKS for “Synthesize 2”, ANS for sweetness, and SCS for bitterness. The electronic nose (PEN3, AIRSENSE, Germany) had a sensor array consisting of ten elements. Each sensor was specialized in detecting certain substances: W1S for methane and related compounds, W3S for methane-aliphatic substances, W5S for polar compounds, nitrogen oxides, and ozone, W6S for hydrogen, W1C for aromatic compounds, W3C for ammonia, aldehydes, and ketones, W5C for aromatic and aliphatic alkanes, W1W for sulfur compounds and terpenes, W2S for alcohols, and W2W for sulfur-containing organic compounds (Sheng et al., 2022). The sensor responses were quantified by electrical resistivity.

### 2.5 In vitro digestion

The *in vitro* simulated digestion assay was performed according to previously reported protocols, with slight modifications incorporated (Sheng et al., 2024; Zheng et al., 2025). The *in vitro* digestion process was carried out using a three-step approach, specifically encompassing oral, gastric, and small intestinal phases. For each digestion step, three parallel replicates were established to ensure reliability. The oral digestion phase was initiated by adding 100 mL of wine to an artificial saliva solution, with the pH adjusted to 6.8 using 1 M NaOH. Subsequently, 1 mL of α-amylase solution (activity ≥25 U) was incorporated into the mixture, which was then incubated at 37 °C with stirring at 100 rpm for 1 min to simulate oral digestion. Following oral digestion, the samples were diluted (*w*/*v*) with simulated gastric fluid (SGF) containing 1 mg/mL pepsin (activity ≥1200 U/g) at pH 1.5. This mixture was stirred at 100 rpm for 2 h at 37 °C to mimic gastric digestion. Finally, simulated intestinal fluid (SIF) containing 4 mg/mL trypsin (activity ≥2500 U/g) at pH 7.4 was added to the samples, and digestion was continued under the same stirring and temperature conditions for an additional 2 h to complete the small intestinal phase. The preparation of simulated fluids such as artificial saliva solution, gastric solution, and small intestinal solution specifically referred to previous references (Sun et al., 2020). Upon completion of the simulated digestion process, the sample solution was immediately frozen and transferred to −20 °C storage for subsequent testing and analysis.

Following digestion, the samples were centrifuged at 8000 rpm for 20 min to collect the supernatants (referred to as micelles). The contents of total phenolics (TP), total anthocyanins (TA), total flavonoids (TF), and total tannins (TT) of in these supernatants and the original wine samples were then measured to assess their bioaccessibility. Additionally, the DPPH free radical scavenging rate and total antioxidant capacity (T-AOC) were evaluated, with all determinations performed according to previously published methods (Sheng et al., 2024). The bioaccessibility of the wine samples was calculated using the following formula:

(1) $$\mathrm{Bioaccessibility}\;\left(\%\right)=\frac{C_{\mathrm{micelles}}}{C_{\mathrm{digesta}}}\times 100$$

where C_micelles_ denotes the total quantity of TP, TF, TT, or TA measured in the digested sample, C_digesta_ signifies the amount of TP, TF, TT, or TA measured in the intestinal phase post-digestion (Sheng et al., 2024).

### 2.6 UHPLC-MS/MS analysis

Metabolomic profiling was conducted utilizing a UPLC-ESI-Q-Orbitrap-MS system, which consisted of a Shimadzu Nexera X2 LC-30 CE UHPLC (Shimadzu, Japan) coupled with a Q-Exactive Plus mass spectrometer (Thermo Scientific, San Jose, USA). For liquid chromatographic separation, samples were analyzed on an ACQUITY UPLC® HSS T3 column (2.1 × 100 mm, 1.8 μm; Waters Corporation, Milford, MA, USA). The specific method referred to previous literature (Zheng et al., 2025). Briefly, the flow rate was set at 0.3 mL/min, with the mobile phase consisting of solvent A (0.1 % formic acid in water) and solvent B (100 % acetonitrile). The gradient program was as follows: initial conditions of 0 % B maintained for 2 min, followed by a linear increase to 48 % B over 4 min, then a further increase to 100 % B over 4 min with this composition held for 2 min. Subsequently, the gradient was reduced to 0 % B within 0.1 min, followed by a 3-min re-equilibration period. Electrospray ionization (ESI) in both positive and negative modes was employed for separate MS data acquisition. The heated electrospray ionization (HESI) source parameters were set as follows: spray voltage at 3.8 kV (positive mode) and 3.2 kV (negative mode); capillary temperature maintained at 320 °C; sheath gas (nitrogen) flow rate at 30 arbitrary units (arb); auxiliary gas flow rate at 5 arb; probe heater temperature at 350 °C; and S-Lens RF level at 50. The instrument was configured to acquire data over an *m*/*z* range of 70–1050 Da for full MS scans. Full MS scans were obtained at a resolution of 70,000 at m/z 200, while MS/MS scans were acquired at a resolution of 17,500 at m/z 200. The maximum injection time was set to 100 ms for MS and 50 ms for MS/MS. For MS2 analysis, the isolation window was set to 2 m/z, and stepped normalized collision energies of 20, 30, and 40 were applied for fragmentation.

### 2.7 Statistical analysis

Statistical analyses were conducted using SPSS 20.0 to assess differences between wine samples. Analysis of variance (ANOVA) was applied, followed by Duncan's multiple comparisons for each parameter. To visually analyze the chemical and sensory differences among wines, bar charts, principal component analysis (PCA), cluster analysis (CA), circos plots, and radar charts were generated using GraphPad Prism 9 and R i386 3.4.2. Multivariate data from metabolic analyses and modeling were handled with R (version 4.3.2) and relevant R packages. Metabolomics analyses were executed on the Bioprofile data analysis cloud platform (http://47.103.152.113/BioCloud/start.php). All experiments were repeated three times.

## 3 Results and discussion

### 3.1 Physicochemical parameters of wines

**Fig. 1:**
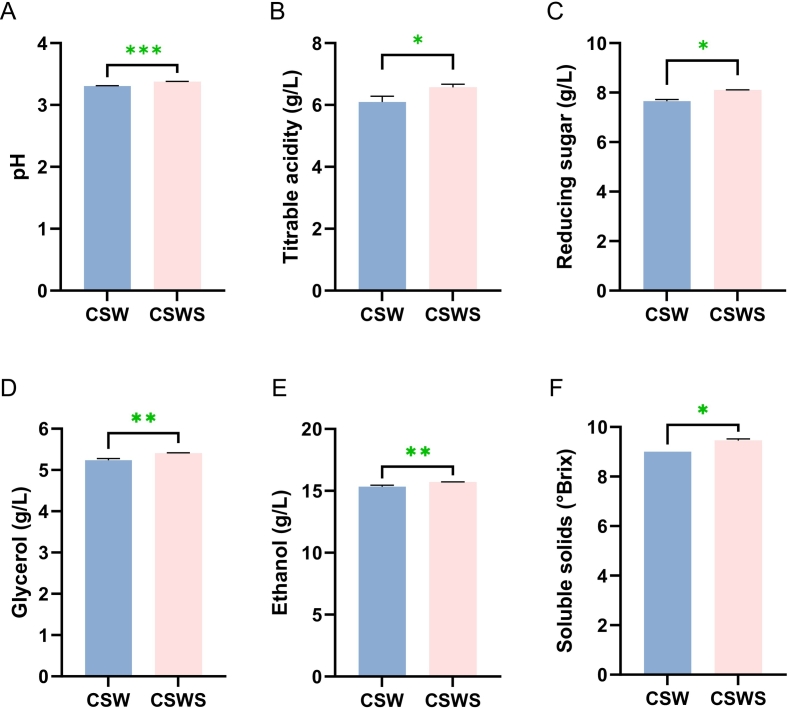
Basic physical and chemical indicators of wines. A: pH in wines; B: Titratable acidity in wines; C: Residual sugar in wines; D: Glycerol in wines; E: Ethanol content in wines; F: Soluble solids in wines; CSW, *Cabernet Sauvignon* wine; CSWS, CSW with *Chardonnay* grape skin residue. All data were presented as means and standard derivations. The results were statistically significant (*p* < 0.05).

Chemical parameters of wine are associated with the presence of pulp and peel residues in the wine (Giuffrida de Esteban et al., 2019; Oliveira et al., 2024). Therefore, this study preliminarily evaluated the impact of adding *Chardonnay* grape skin residue on the basic physicochemical indices of *Cabernet Sauvignon* wine. The results of the single-factor analysis showed that there were significant differences (*p* < 0.05) in parameters such as soluble solids, pH, titratable acidity, residual sugar, and glycerol between the wines with and without the addition of *Chardonnay* grape skin residue. This further demonstrated the influence of grape skin residue on the basic characteristics of *Cabernet Sauvignon* red wine (Fig. 1). Notably, the pH values of samples ranged from 3.30 to 3.38, and CSW and CSWS showed a remarkable difference (*p* < 0.01) (Fig. 1A). The range of titratable acidity was 6.00 to 6.56 g/L, and the titratable acidity content in CSWS was significantly higher than that in CSW (p < 0.05) (Fig. 1B). The contents of the reducing sugar and glycerol of samples were 7.60 to 8.10 g/L and 5.23 to 5.41 g/L, respectively (p < 0.05) (Fig. 1C and D). The range of soluble solids values of samples ranged from 9.00°Brix in CSW to 9.45°Brix in CSWS (p < 0.05) (Fig. 1F). It was noteworthy that the adding of *Chardonnay* grape skin residue had effectively increased the alcohol content in the CSWS, with the resulting CSWS wine having an alcohol content of 15.7 g/L (Fig. 1E). Overall, the incorporation of *Chardonnay* grape skin residue was beneficial for enhancing the fundamental physicochemical properties of wine.

### 3.2 Determination of organic acids in wines

**Fig. 2:**
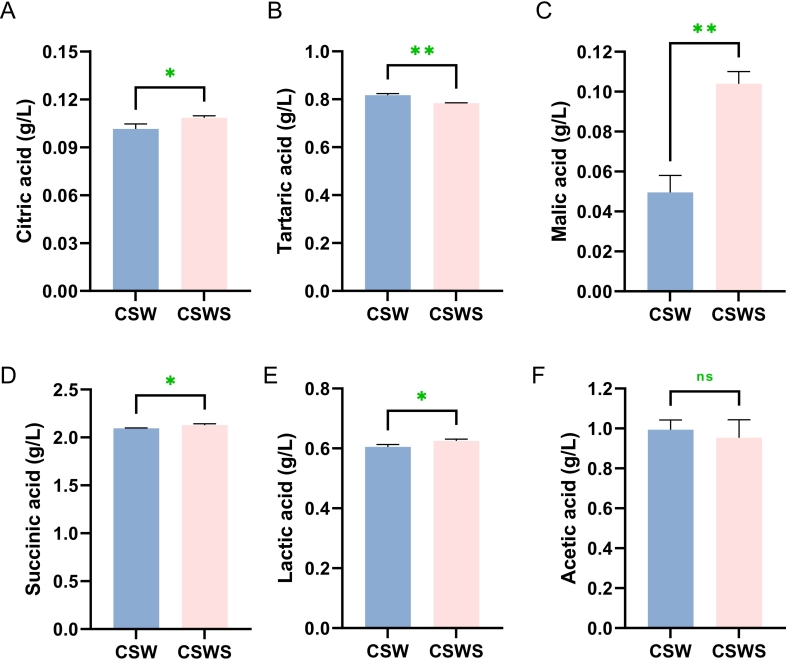
The profiles of organic acids of wines. A, B, C, D, E and F: Citric acid/tartaric acid/malic acid/succinic acid/lactic acid/acetic acid in wines. CSW, *Cabernet Sauvignon* wine; CSWS, CSW with *Chardonnay* grape skin residue. All data were presented as means and standard derivations. The results were statistically significant (*p* < 0.05).

In addition, grape skin residue contains some substances with special biological activities, such as organic acids like tartaric acid and malic acid, which play a certain role in regulating the flavor and taste of wine (Yang et al., 2024). Among all organic acids, the addition of *Chardonnay* grape skin residue significantly increased the contents of citric acid, malic acid, succinic acid, and lactic acid, indicating that the pomace contains a certain amount of these acids (Fig. 2). Succinic acid, lactic acid, citric acid, malic acid had concentration ranges of 2.09 to 2.13 g/L, 0.60 to 0.63 g/L, 0.10 to 0.11 g/L and 0.05 to 0.10 g/L respectively, and succinic acid showed the greatest abundance (Fig. 2A, C, D, F). Notably, after the addition of *Chardonnay* grape skin residue, the contents of tartaric acid and acetic acid in the wine decreased, from 0.82 g/L and 0.99 g/L to 0.78 g/L and 0.95 g/L, respectively (Fig. 2B and F). In summary, the addition of *Chardonnay* grape skin residue is capable of increasing the content of most organic acids in *Cabernet Sauvignon* wine.

### 3.3 Assessment of wines using electronic senses

**Fig. 3:**
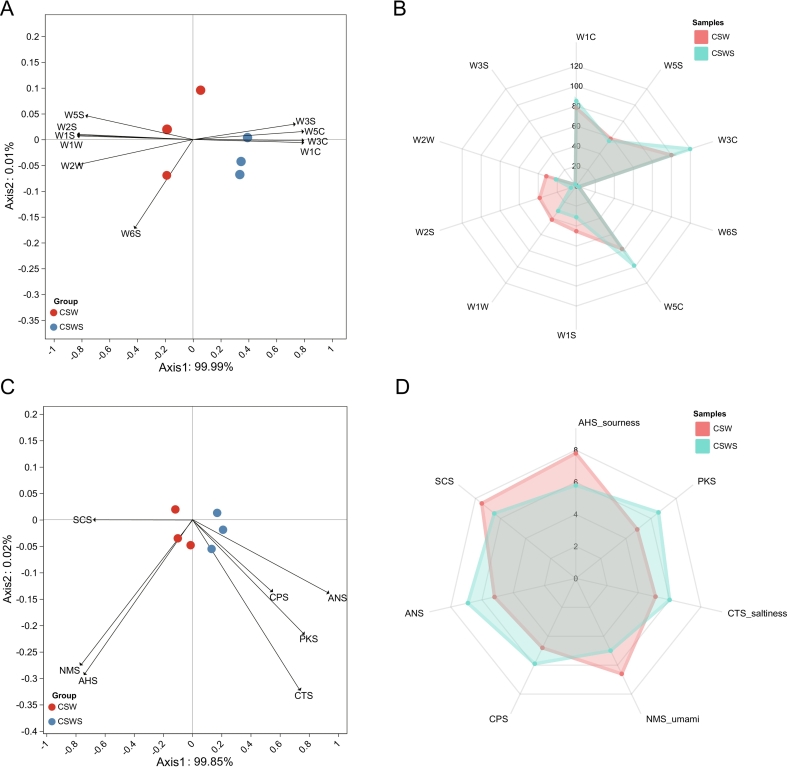
Electronic sensory evaluation of wines. A and B: PCA of e-nose and e-tongue in wines. C and D: E-nose and e-tongue data radar chart of taste quality in wines. CSW, *Cabernet Sauvignon* wine; CSWS, CSW with *Chardonnay* grape skin residue. All data were presented as means and standard derivations. The results were statistically significant (p < 0.05).

As analytical tools that simulate the human olfactory and gustatory systems, the electronic nose and electronic tongue are equipped with different types of sensors. These sensors can respond to the volatile substances and taste substances in wine, and they can be used for quality grading and quality control of wine. Using MOS chemical sensors, there was a statistically significant difference in the response values between the two groups of wines (*p* < 0.05), indicating that the addition of *Chardonnay* grape skin residue had a significant impact on the aroma of *Cabernet Sauvignon* wine (Fig. 3A). In the two groups of wines, the total response value of the CSWS group (473) was higher than that of the CSW group (458) (Fig. 3B). Secondly, according to the results of Principal Component Analysis (PCA), the first two principal components (PCs) contributed 100 % to the total variance. Among them, PC1 accounts for 99.99 % of this variance, and PC2 accounts for 0.01 % (Fig. 3A). The correlation between the sensors and sample distribution showed that the samples in the CSWS group clustered on the positive half-axis of PC1, while most of the samples in the CSW group scattered on the negative half-axis of PC2. Notably, both groups exhibited a distribution across the positive and negative halves of the PC2 axis. Nevertheless, the considerable gap between the aggregated regions of the CSW and CSWS groups demonstrated that the inclusion of *Chardonnay* grape skin residue significantly altered the aroma characteristics of *Cabernet Sauvignon* wine. Furthermore, among the E-nose sensors, W1C, W3C, W5C, and W3S were distributed on the positive half-axis of PC1, while W5S, W2S, W1S, W1W, and W2W were located on the negative half-axis of PC2. The positional correlation analysis revealed that, although the CSW group exhibited a strong positive correlation with most of the sensors, the CSWS group possessed a more diverse and abundant profile of aromatic compounds compared to the CSW group. For instance, the CSWS group showed a significant positive correlation with the W1C, W3C, and W5C sensors. Due to their close positional relationship, this group generated a greater variety of aromas. In contrast, the CSW group was predominantly positively correlated with sensors such as W1W, W1S, W2S, W5S, and W5C. Consequently, distinct discrepancies in olfactory characteristics emerged between the two wine groups.

Subsequently, the PCA analysis of the electronic tongue revealed distinct differences in the taste profiles of wines from the CSW and CSWS groups. PC1, with a contribution rate of 99.85 %, exerted a dominant influence, while PC2 contributed only 0.02 % (Fig. 3C). The CSWS group showed significant responses to sensors such as ANS, CPS, PKS, and CTS. In contrast, the CSW group exhibited higher response intensities to sensors like NMS, SCS, and AHS. Although there were differences in the response intensities of the remaining sensor probes, these differences were not statistically significant. The results indicated that the wines in the CSWS group were characterized by sweetness and saltiness, while those in the CSW group were mainly featured with umami, sourness or bitterness, suggesting that the samples of the two groups had unique taste profiles (Fig. 3D). By adding *Chardonnay* grape skin residue, the bitterness and sourness of the wine gradually decreased. Interestingly, two comprehensive attributes (PKS, CPS) of the CSWS group were significantly higher than that of the CSW group. These results demonstrated that the addition of grape skin residue contributed to the improvement of the wine's taste and aroma. The overall flavor imparted by the wine after aging could further enhance its complexity and layers. In conclusion, the CSWS group exhibited more significant differences in both taste and aroma aspects, which may be attributed to the increase in aromatic compounds and the decrease in compounds causing astringency and bitterness. To further clarify the differential aroma components, subsequently, we carried out an analysis of the aroma substances using HS-SPME/GC–MS.

### 3.4 Volatile aroma profiles of wines

**Fig. 4:**
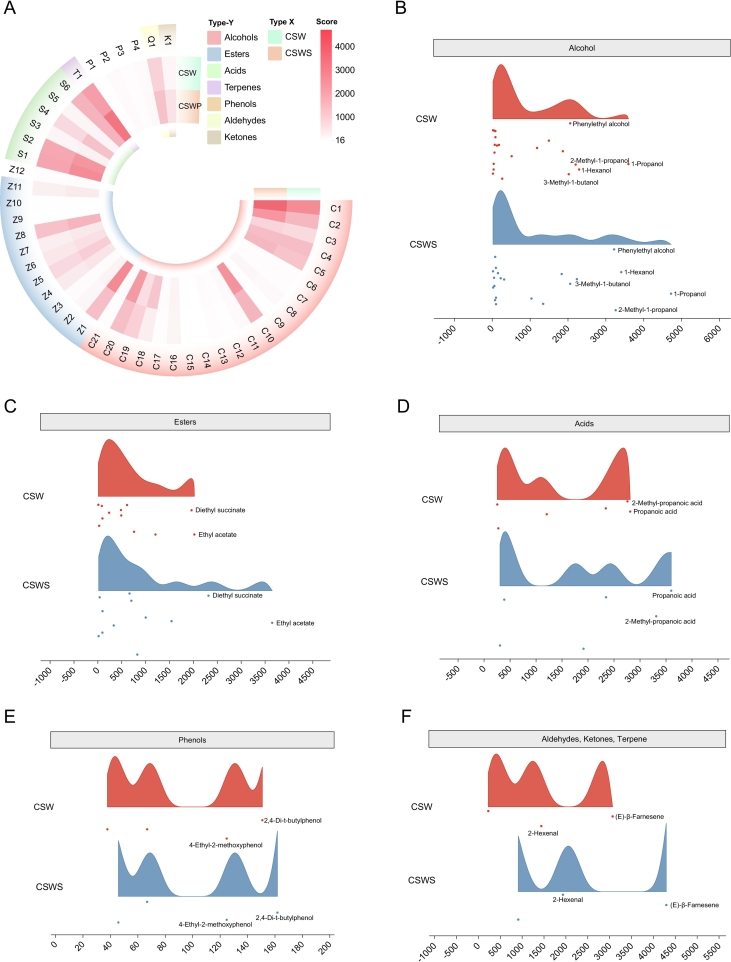
Aroma compound profiles in wines. A: The heatmap of aroma compounds; B, C, D, E and F: Cloud and rain plot of alcohols, esters, acids, phenols and aldehydes/quinones/terpenoids. CSW, *Cabernet Sauvignon* wine; CSWS, CSW with *Chardonnay* grape skin residue. All data were presented as means and standard derivations. The results were statistically significant (p < 0.05).

A total of 46 volatile compounds were identified in the two groups of wines (Table 1). The most diverse aroma compounds in the wines were esters and alcohols, followed by acids, phenols, terpenes and aldehydes (Fig. 4A). Details of the chromatograms and mass spectra of volatile compounds can be found in Figs. S1-S3. It was worth noting that among the volatile aroma compounds of the wines, a total of 21 alcohols and 12 esters were detected. The results indicated that the wines in the CSWS group had the highest cumulative aroma abundance, followed by those in the CSW group. Next, we used heat maps to display the differences in the wine aroma profiles and found that significant changes occurred in alcohols, esters, and terpenes among the 46 aroma compounds. Alcohols such as C1 (1-Propanol), C2 (2-Methyl-1-propanol), C10 (1-hexanol), C18 (1-hexadecanol), C20 (phenylethyl alcohol); esters such as Z1 (ethyl acetate), Z5 (ethyl caprylate), Z8 (diethyl succinate); and terpenes such as T1 ((E)-β-farnesene) showed higher intensities in the wines of the CSWS group (Fig. 4A, B, C, F). Intriguingly, the concentrations of C3 (1-butanol), C4 (4-methyl-2-pentanol), C5 (3-methyl-1-butanol), C8 (3-methyl-1-pentanol), C16 (nerol), C19 (benzyl alcohol), Z2 (ethyl butanoate), Z4 (ethyl hexanoate), Z5 (ethyl caprylate), Z6 (ethyl 3-hydroxybutyrate), Z7 (ethyl caprate), Z11 (isoamyl acetate), S1 (propanoic acid), S2 (2-methyl-propanoic acid), S4 (octanoic acid), S6 (benzoic acid), Q1 (2-hexenal) and K1 ((*Z*)-oak lactone) were generally consistent in the wines of the two groups, jointly forming the basic flavor framework of the wine (Fig. 4A). Evidently, the addition of *Chardonnay* grape skin residue significantly accentuated the distribution of alcohol, as well as flavors such as pineapple, strawberry, fruity notes (C1, C2, Z1, Z5, Z8, T1, Q1, Z6, C19), herbaceous undertones (C10, S6), floral aromas (Z5, T1), rose scents (C18, C20, Z11, C16), and cheesy flavors (S2, S4, S5) (Fig. 4A). Subsequently, we further demonstrated the differential changes in the typical compounds of the single aroma characteristics between the two groups of wines by using a cloud and rain plot. Among the alcohols, 1-propanol, 1-hexanol, phenylethyl alcohol, 2-methyl-1-propanol, and 3-methyl-1-butanol were typical components (Fig. 4B). For esters, typical compounds included ethyl acetate and diethyl succinate (Fig. 4C). As for acids, propanoic acid and 2-methyl-propanoic acid were typical compounds (Fig. 4D). For phenols, 2,4-di-t-butylphenol and 4-ethyl-2-methoxyphenol, for terpenes, (E)-β-farnesene, and for aldehydes, 2-hexenal and the like were all typical compounds (Fig. 4E and F). In conclusion, the addition of *Chardonnay* grape skin residue improved the volatile chemical flavor profiles of the wines.

**Table 1:** Aromatic compounds contents in different wines.

No.	Compounds	CSW (μg/L)	CSWS (μg/L)	Odor	Threshold (μg/L)
**Alcohols**					
C1	1-Propanol	3603 ± 404^a^	4728 ± 177^a^	Alcohol, ripe, fruit	30,600
C2	2-Methyl-1-propanol	2209 ± 311^a^	3266 ± 1231^a^	Alcoholic	75,000
C3	1-Butanol	1190 ± 34^a^	1352 ± 161^a^	Alcoholic	1500
C4	4-Methyl-2-pentanol	2066 ± 120^a^	2066 ± 210^a^	Almond, toasted	50,000
C5	3-Methyl-1-butanol	1872 ± 127^a^	2242 ± 481^a^	Solvent	60,000
C6	(*Z*)-3-Hexen-1-ol	65 ± 6^a^	81 ± 32^a^	Green, bitter, fatty	1000
C7	4-Methyl-1-pentanol	67 ± 5^a^	81 ± 32^a^	Almond, toasted	50,000
C8	3-Methyl-1-pentanol	186 ± 15^a^	231 ± 42^a^	Vinous, herbaceous, cacao	50,000
C9	1-Octen-3-ol	44 ± 4^a^	55 ± 15^a^	Mushroom	1
C10	1-Hexanol	7302 ± 659^b^	9414 ± 1841^a^	Herbaceous, grass, woody	110
C11	(E)-3-Hexen-1-ol	133 ± 13^a^	159 ± 34^a^	Green	400
C12	(Z)-2-Hexen-1-ol	46 ± 6^b^	64 ± 12^a^	Green grass, herb	400
C13	1-Heptanol	92 ± 6^a^	106 ± 18^a^	Lemon, orange, copper	200
C14	α-Terpineol	27 ± 1^a^	27 ± 8^a^	Floral, sweet	1000
C15	Linalool	28 ± 1^a^	33 ± 6^a^	Citrus, floral, sweet, grape-like	15
C16	Nerol	271 ± 7^a^	321 ± 71^a^	Rose, lime	400
C17	1-Decanol	73 ± 3^a^	107 ± 48^a^	Sweet, fatty	400
C18	1-Hexadecanol	520 ± 19^c^	1841 ± 210^a^	Rose scent	nf
C19	Benzyl alcohol	1503 ± 120^a^	1043 ± 144^a^	Sweet, fruity	200,000
C20	Phenylethyl alcohol	5027 ± 370^a^	6225 ± 122^a^	Floral, rose, honey	10,000
C21	(Z,E)-farnesol	90 ± 10^b^	104 ± 2^a^	Floral	2400
**Esters**					
Z1	Ethyl acetate	2026 ± 557^a^	3655 ± 51425^a^	Pineapple, fruity, solvent	12,000
Z2	Ethyl butanoate	241 ± 22^b^	338 ± 25^a^	Burnt, cheese	600
Z3	Isobutyl isovalerate	102 ± 9^a^	103 ± 15^a^	Strawberry or apple aroma	1400
Z4	Ethyl hexanoate	617 ± 74^a^	831 ± 587^a^	Pineapple aroma	100
Z5	Ethyl caprylate	1212 ± 201^a^	1548 ± 739^a^	Fruity, flowery, sweet almond, soap, candle	600
Z6	Ethyl 3-hydroxybutyrate	764 ± 82^a^	1007 ± 134^a^	Pineapple, strawberry, tea, honey	1000
Z7	Ethyl caprate	495 ± 85^a^	707 ± 235^a^	Soap, candle	510
Z8	Diethyl succinate	1967 ± 195^a^	2321 ± 306^a^	Fruity	1200
Z9	Ethyl benzoate	16 ± 2^a^	22 ± 6^a^	Fruity	56
Z10	Phenethyl acetate	33 ± 3^a^	43 ± 7^a^	Pleasant, floral	250
Z11	Isoamyl acetate	490 ± 41^b^	668 ± 424^a^	Banana, fruity, sweet	160
Z12	Ethyl 9-decenoate	89 ± 18^b^	101 ± 32^a^	Rose	100
**Acids**					
S1	Propanoic acid	2817 ± 664^a^	3606 ± 664^a^	Pungent, rancid, soy	8100
S2	2-Methyl-propanoic acid	2764 ± 513^a^	3317 ± 1314^a^	Cheese, fatty	200,000
S3	3-Methyl-butanoic acid	280 ± 12^b^	394 ± 10^a^	Rancid, acidic	3000
S4	Octanoic acid	1212 ± 157^b^	1919 ± 212^a^	Fatty, rancid	10,000
S5	n-Decanoic acid	260 ± 27^b^	312 ± 2^a^	Fatty, rancid	6
S6	Benzoic acid	2348 ± 27^b^	2353 ± 94^a^	Light and pleasant fragrance	nf
**Phenols**					
P1	Phenol	38 ± 3^b^	46 ± 28^a^	Flavor	40,000
P2	4-Ethyl-2-methoxyphenol	125 ± 1^a^	125 ± 1^a^	Medicine, wood, clove, smoky	33
P3	4-Ethyl-phenol	67 ± 3^a^	67 ± 6^a^	Shoe polish, baking	610
P4	2,4-Di-t-butylphenol	151 ± 7^a^	162 ± 31^a^	Alkylphenol odor	500
**Terpenes**					
T1	(E)-β-Farnesene	3074 ± 556^c^	4301 ± 774^a^	Citrus green, floral	nf
**Aldehydes**					
Q1	2-Hexenal	1445 ± 189^a^	1939 ± 117^a^	Aroma of fresh fruit and green leaves	nf
**Ketones**					
K1	(Z)-oak lactone	232 ± 84^c^	918 ± 97^a^	Coconut, vanilla	nf

Note: Data were shown as mean ± standard deviation of three sets of replicated trials, each set of data was analyzed by one-way ANOVA to mark significant differences (p < 0.05). nf means the threshold value of the compound is unknown.

### 3.5 Profile of non-volatile metabolites in wines

The research on non-targeted metabolites can comprehensively reflect the components of wine, thus revealing the mechanism of its quality formation (Welke et al., 2024). Therefore, we further investigated the differences in non-target metabolites between the two groups of wines. In this study, a total of 914 non-volatile metabolites were identified. Among them, 120 non-volatile metabolites were detected in the negative ion mode, and 764 metabolites were detected in the positive ion mode (Table S1). The metabolite chromatograms are detailed in Figs. S4-S7, and the original files of the mass spectra of the metabolites are provided in Supplementary material 3, Supplementary material 4, Supplementary material 5, Supplementary material 6, Supplementary material 7, Supplementary material 8, Supplementary material 9, Supplementary material 10. Subsequently, we used a clustering heatmap to explore the variation characteristics of non-volatile metabolites in the two groups of wine samples. We selected the top 50 differential compounds with high variable importance in the projection (VIP) values for clustering analysis (Fig. S8). All non-volatile metabolites were mainly divided into two clusters. In the first cluster, we observed that a variety of compounds were significantly enriched in CSW, including indole-3-ethanol, myrsinone, sarcosine, and 5-hydroxy-4-oxo-L-norvaline. In the second cluster, CSW was significantly enriched with a number of compounds such as 3-aminopicolinaldehyde, Di-N-propylamine, leucylalanine, 7,9-dimethoxy-6-(4-methoxyphen), LPC (P-16:0), 3-hydroxy-4,5-dimethyl-2(5H)-furanone, carveol, valine, and imidazole lactate (Fig. S8). However, in the CSWS group, the contents of indole-3-ethanol, myrsinone, sarcosine, and 5-hydroxy-4-oxo-L-norvaline in the first cluster decreased, while 3-hydroxyadipic acid 3,6-lactone and PC (16:0/18:1) were significantly increased (Fig. S8). In the second cluster, the concentrations of compounds such as 3-aminopicolinaldehyde, LPC(P-16:0), 3-hydroxy-4,5-dimethyl-2(5H)-furanone, and leucylalanine in the CSWS group decreased significantly, while the concentrations of compounds such as PC (18:1/16:0), 16-amino-1-cyclohexylhexadecan-1-one, narcissin, rutin, isoleucylaspartate, and chrysin 7-O-beta-gentiobioside increased significantly.

The changes in the contents of PC (18:1/16:0) and LPC (P-16:0) indicate that the addition of grape skin residue may have affected the alteration of the phospholipid pathway in microorganisms (Yang et al., 2024). The increase in the contents of 16-amino-1-cyclohexylhexadecan-1-one, narcissin, rutin and isoleucylaspartate indicated that after the addition of grape skin residue, a series of complex chemical reactions had taken place during the aging process of the wine. Among these, chrysin 7-O-beta-gentiobioside, narcissin, and rutin all belong to flavonoid compounds and possess strong antioxidant properties. The increase in their contents in the wine will further enhance the wine's antioxidant capacity, thereby contributing to the stability of the wine during the aging process (Tzachristas et al., 2020). It is worth noting that as an amino acid, the increase in the content of isoleucylaspartate might indicate that the Maillard reaction had taken place in the wine. The Maillard reaction typically gives rise to a variety of compounds with unique aromas and flavors, such as aldehydes, pyrazines, and furans (Liu et al., 2022). These compounds endow the wine with aromas like roasted scent, caramel aroma, and chocolate aroma, enriching the aroma composition of the wine and enhancing its sensory quality. However, pyrazines and furans were not detected among the volatile compounds, which suggests that the contents of these compounds might be below the detection limit. In conclusion, the addition of *Chardonnay* grape skin residue can endow *Cabernet Sauvignon* wine with metabolites that possess special flavors or biological activities.

### 3.6 *In vitro* digestion

#### 3.6.1 Digestive characteristics of wines

**Fig. 5:**
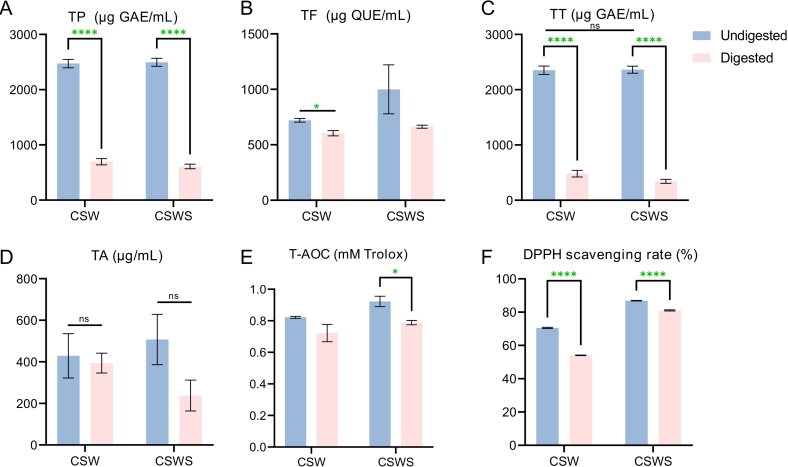
The digestive characteristics of wines. A, B, C and D: Effects of total phenolics (TP), total flavonoids (TF), total tannins (TT), and total anthocyanin (TA) content of wines; E and F: Effects of total antioxidant capacity (T-AOC) and DPPH of wines. CSW, *Cabernet Sauvignon* wine; CSWS, CSW with *Chardonnay* grape skin residue. All data were presented as means and standard derivations. The results were statistically significant (p < 0.05).

Moderate alcohol consumption has health benefits such as promoting intestinal peristalsis, improving digestive system function, and preventing cardiovascular diseases (Taladrid et al., 2023). Since polyphenolic substances such as tannins and anthocyanins in *Cabernet Sauvignon* wine mainly affect the astringency and color stability of the wine, we compared the effects of adding CGSR on these aspects. Firstly, we observed that by adding *Chardonnay* grape skin residue, the contents of TP, TF, TA, and TT in the CSWS group were increased, from 2473 μg GAE/mL, 721 μg QUE/mL, 429 μg/mL and 2353 μg GAE/mL to 2496 μg GAE/mL, 1000 μg QUE/mL, 507 μg/mL and 2363 μg GAE/mL, respectively (Fig. 5A, B, C, and D). As expected, the addition of CGSR did not significantly increase the contents of TT and TA. Subsequently, the experiments on the antioxidant activities of the wines in the two groups demonstrated that the T-AOC and DPPH scavenging rate of the wines in the CSWS group were significantly higher than those in the control group (CSW) (Fig. 5E, F). It is worth noting that after adding CGSR, the total antioxidant capacity of the wine increased by 17.7 %. These results indicated that the addition of grape skin residue helped to enhance the nutritional value of the wine, including the increase in the contents of bioactive substances such as TF, TP, TA, and TT, as well as the enhancement of antioxidant activity (Fig. 5).

Next, by simulating the conditions of the oral cavity, stomach, and intestine, we further explored the *in vitro* digestion characteristics and bioaccessibility of the two groups of wines. The INFO-equigest standardized *in vitro* digestion procedure was adopted to investigate the changes in TP, TF, TA, and TT of the two groups of wines before and after *in vitro* digestion (Fig. 5) (Sheng et al., 2024). Since the wine, after being digested in the oral cavity and stomach, is transferred to the small intestine for subsequently absorbed, we tested various digestion index parameters of the samples after the final absorption in the small intestine. Overall, during the *in vitro* digestion process, both wine groups demonstrated excellent absorption property for TP and TT, with bioaccessibility ranging from 69.2 % to 75.6 % and 79.5 % to 85.5 %, respectively (Fig. 5A, C). In contrast, the bioaccessibility of TF and TA was 16.1–33.7 % and 8.2–53.1 %, respectively, which were significantly lower than those of TP and TT. These findings strongly suggested that approximately 72.4 % of polyphenols, 82.5 % of the tannins, 24.9 % of flavonoids and 30.6 % of anthocyanin present in wine were absorbed during digestion in the oral cavity, stomach, and small intestine. These results indicated that TP and TT in wine were likely to be more readily absorbed and utilized by the human digestive system, thereby enabling them to exert their potential health benefits, such as antioxidant and anti-inflammatory effects. It is worth noting that the bioaccessibility of TP and TT in the CSWS group increased by 9.2 % and 7.5 % respectively compared with that in CSW, while the bioaccessibility of TF and TA increased by 109 % and 549 % respectively (Fig. 5). In addition, after digestion in the small intestine, the total antioxidant capacity and DPPH scavenging rate of the CSWS group were both higher than those of the CSW group, which was consistent with the values of TT, TF, and TP (Fig. 5). After *in vitro* digestion, the T-AOC and DPPH scavenging rate of the CSWS group decreased from 0.92 mM Trolox and 86.9 % to 0.79 mM Trolox and 81.1 %, respectively (Fig. 5E and F). Typically, antioxidant capacity exhibits a significant correlation with the levels of TP and TF (Xi et al., 2024). Based on the findings of this research, the enhanced antioxidant capacity observed in the CSWS group can likely be attributed to variations in polyphenol content.

#### 3.6.2 Changes in wine metabolites after *in vitro* digestion

**Fig. 6:**
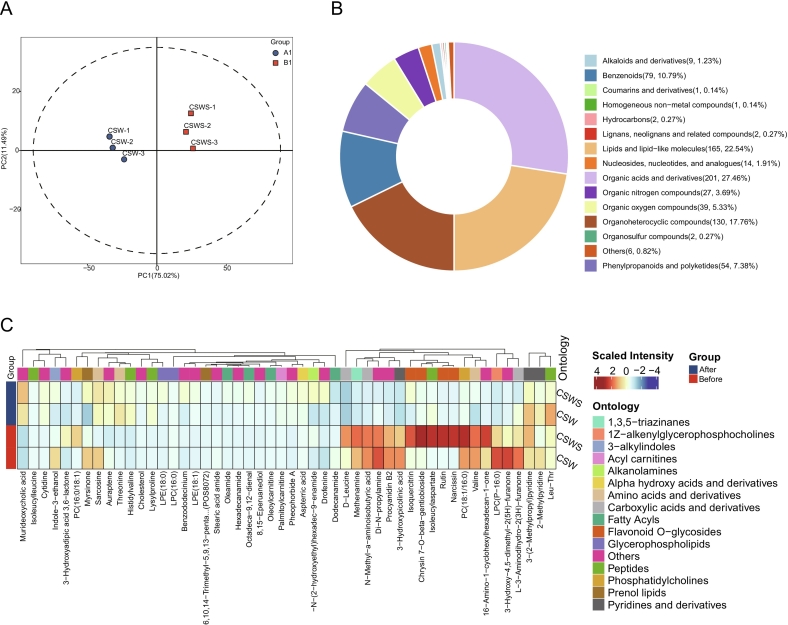
Changes of metabolites in wines before and after *in vitro* simulated digestion. A: PCA for differential metabolites in wines; B: Classification ring diagram of differential metabolites in wines; C: The clustering heatmap of differential metabolites in wines; Before, before *in vitro* simulated digestion; After, after *in vitro* simulated digestion. CSW, *Cabernet Sauvignon* wine; CSWS, CSW with *Chardonnay* grape skin residue. All data were presented as means and standard derivations.

Next, we characterized the impact of *Chardonnay* grape skin residue on *Cabernet Sauvignon* wine by detecting the differences in metabolites of the two groups of wines before and after *in vitro* digestion. Based on PCA, the variances of PC1 and PC2 were 75.02 % and 11.49 %, respectively, and they accounted for 86.51 % of the total variance (Fig. 6A). The results showed that there was no difference within each of the two groups of samples, but there was a difference between the groups, indicating that the metabolites in the wine had changed significantly before and after digestion. The ring-shaped pie chart illustrated the distribution of various compounds, covering a total of 15 categories including organic acids and their derivatives, lipids and lipid-like molecules. Among them, organic acids and their derivatives, lipids and lipid-like molecules, organoheterocyclic compounds and benzenoids had relatively large proportions, reaching 27.46 %, 22.54 %, 17.76 %, and 10.79 % respectively (Fig. 6B). In contrast, coumarins and their derivatives, homogeneous non-metal compounds, *etc.* accounted for extremely small proportions, merely 0.14 %. Notably, in the preliminary experiment (Section 3.6.1), the TT in CSW and CSWS showed no difference, which was consistent with the metabolomic results for tannins (see Supplementary Table S1 for details). Given that wine astringency is caused by multiple chemicals, we focused on changes in phenolic acids in addition to tannin, the key component (Fig. S9). The results indicated that gallic acid, caffeic acid, and syringic acid in CSW and CSWS did not change significantly (Fig. S9A, S9B, and S9C). Although p-coumaric acid in CSWS decreased significantly compared with that in CSW, this might not lead to a notable change in astringency (Fig. S9D). Thus, we speculate that astringency may not have changed significantly during fermentation.

Furthermore, a clustering heatmap was used to demonstrate differences in metabolite changes before and after *in vitro* digestion. The findings indicated that after *in vitro* digestion, the metabolites in both groups of wine samples exhibited remarkable alterations (Fig. 6C). Notably, substances such as D-leucine, methenamine, isoquercitrin, PC (16:0/18:1), chrysin 7-O-β-gentiobioside, isoleucylaspartate, rutin, and narcissin, which were significantly enriched in CSWS, showed a remarkable decline in their contents after digestion. In addition, after digestion, the contents of murideoxycholic acid and cytidine in the two groups of samples increased. Among them, as a bile acid, murideoxycholic acid can emulsify fats, promote fat absorption, regulate bile secretion and excretion, *etc.* (Lv et al., 2023), which may explain the reasons for the decrease in the contents of PC and LPC in the wine samples (Fig. 6C). Unlike the CSWS group, the contents of indole-3-ethanol, myrsinone and sarcosine decreased in the CSW group, while the contents of 3-(2-methylpropyl) pyridine and Leu-Thr increased. Among these substances, myrsinone, a phenolic compound endowed with remarkable antioxidant properties, plays a crucial role in safeguarding the components within wine against oxidation (Thoa & Son, 2024). By doing so, it effectively stabilizes both the color and flavor profile of the wine, ultimately contributing to an extended shelf life. In addition, indole-3-ethanol has aromas similar to floral and fruity, which can add layers to the complexity of the wine's aroma (Valera et al., 2021).

#### 3.6.3 Functional analysis of differential metabolites in CSWS after *in vitro* digestion

**Fig. 7:**
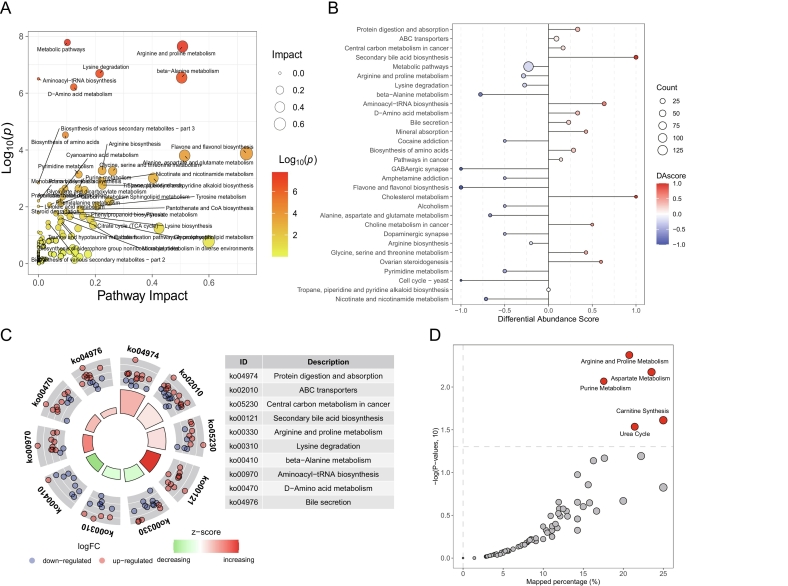
Analysis of pathway enrichment and function of differential metabolites in the CSWS group before and after *in vitro* digestion. A: Pathway impact diagram of the CSWS croup after digestion *vs.* before digestion; B: Differential abundance scores plot of differential metabolites in wines; C: KEGG pathway circular diagram of the top 10 significantly different metabolites. In the left plot, from the outer circle to the inner circle in turn are the numbers of the functions, the metabolites corresponding to the functions (one dot represents one metabolite, with blue indicating down-regulation and red indicating up-regulation), and the z-score values of the functions; D: Functional annotation and enrichment analysis of differential metabolites in the Small Molecule Pathway Database (SMPDB). The fill color of the bubble ranges from deep red to light, indicating an increase in the *p*-value and a decrease in significance. The results were statistically significant (p < 0.05). All data were presented as means and standard derivations. (For interpretation of the references to color in this figure legend, the reader is referred to the web version of this article.)

In organisms, different metabolites coordinate with each other to carry out a series of biochemical reactions, thereby fulfilling their biological functions. The correlations of metabolites indicated that indole-3-ethanol had a strong positive correlation with 3-hydroxyadipic acid 3,6-lactone and stearic acid amide; methenamine had a strong positive correlation with L-3-aminodihydro-2(3H)-furanone, valine and isoleucylaspartate; and stearic acid amide had a strong positive correlation with 3-hydroxyadipic acid 3,6-lactone (Fig. S10). Moreover, there was a strong negative correlation between valine and stearic acid amide. Metabolite network pathway enrichment analysis revealed that, following digestion, several pathways including arginine and proline metabolism, beta-alanine metabolism, flavone and flavonol biosynthesis, and metabolic pathways were notably changed. Significantly, amino acid metabolism-related metabolites accounted for the largest proportion among them (Fig. 7A, Fig. S11). KEGG functional enrichment analysis showed that among various pathways, metabolism stood out with the most remarkable influence and the largest quantity of differential metabolites, while biological systems, human diseases, environmental information processing, *etc.*, followed (Fig. S12A, Fig. S12B, Fig. 7B).

Among all the pathways, the top ten significantly altered enriched pathways mainly include protein digestion and absorption, ABC transporters, central carbon metabolism in cancer, secondary bile acid biosynthesis, arginine and proline metabolism, lysine degradation, beta-alanine metabolism, aminoacyl-tRNA biosynthesis, D-amino acid metabolism, and bile secretion (Fig. 7C). The findings revealed that metabolites post-digestion primarily resulted in the upregulation of several pathways, namely secondary bile acid biosynthesis, cholesterol metabolism, aminoacyl-tRNA biosynthesis, and ovarian steroidogenesis. In contrast, a series of other pathways, including flavone and flavonol biosynthesis, GABAergic synapse, beta-alanine metabolism, nicotinate and nicotinamide metabolism, and alanine, aspartate and glutamate metabolism, experienced downregulation (Fig. 7B, Fig. S13A, Fig. S13B). The correlation analysis between metabolites and pathways showed that the secondary bile acid biosynthesis metabolic pathway was mainly associated with 11 significantly upregulated metabolites, including taurocholic acid, cholic acid, chenodeoxycholic acid (Fig. 7C, Fig. S14). The D-amino acid metabolism pathway was upregulated, which was mainly associated with the significant upregulation of serine, phenylalanine, threonine, methionine, lysine, and arginine (Fig. S14). Metabolites such as succinic acid, fumaric acid, malic acid, arginine, glutamic acid *etc.* were mainly associated with the central carbon metabolism in cancer pathway (Fig. S14). The beta-alanine metabolism pathway showed a significant downregulation, which was mainly associated with eight significantly downregulated metabolites, such as spermidine, beta-alanine, aspartate, and others (Fig. 7C, Fig. S14). Generally, arginine and proline metabolism, aspartate metabolism, purine metabolism, carnitine synthesis, urea cycle, *etc.* are associated with specific diseases. For instance, abnormal arginine and proline metabolism is linked to cardiovascular diseases and certain types of cancer (Ling et al., 2023). Disorders in purine metabolism can give rise to gout and some immune diseases (Huang et al., 2021). Urea cycle disorders can lead to metabolic diseases such as hyperammonemia (Ribas et al., 2022). In this study, these pathways were significantly enriched (Fig. 7D, Fig. S15). Therefore, analyzing these enriched pathways is beneficial for exploring the pathogenesis of diseases, uncovering potential biomarkers, and determining therapeutic targets, thereby develop a functional dry red wine with both dietary therapy and health care functions.

## 4 Conclusion

In conclusion, this study demonstrated the effects of adding CGSR on the chemical, sensory, and nutritional properties of *Cabernet Sauvignon* wine. The results of sensory analysis showed that after adding CGSR, the bitterness, umami, and sourness of the wine were significantly reduced, while the fruity and floral aromas were significantly enhanced. Volatile compounds were significantly enriched with substances such as 1-propanol, phenylethyl alcohol, ethyl acetate, diethyl succinate, and (E)-β-farnesene, which endowed the wine with floral, rose, fruity, and pineapple-like aroma components. The antioxidant capacity and bioavailability of the wine were significantly enhanced, with the bioaccessibility of TP and TA increasing by 9.2 % and 7.5 %, respectively. Based on non-targeted metabolomics, the contents of amino acids, flavonoids, and organic acids changed significantly, such as D-leucine, isoquercitrin, isolaspartic acid, rutin, narcissin *etc.* Furthermore, after digestion, pathways related to human metabolism, such as arginine and proline metabolism, aspartic acid metabolism, and purine metabolism, were significantly enriched. In the future, it will be possible to conduct in-depth analyses of potential biomarkers and identify therapeutic targets for human diseases. Therefore, this study provides a promising new strategy for improving the quality of *Cabernet Sauvignon* wine and, by analyzing the functional metabolic pathways enriched by key metabolites, offers reference value for the design of functional wines with dietary therapy and health-care effects.

## CRediT authorship contribution statement

**Xiaochun Zheng:** Writing – original draft, Visualization, Software, Resources, Project administration, Methodology, Data curation, Conceptualization. **Jie Sheng:** Writing – review & editing, Visualization, Supervision, Software, Data curation, Conceptualization. **Yu Chen:** Writing – review & editing, Software. **Bin Wang:** Writing – review & editing, Funding acquisition. **Xuewei Shi:** Writing – review & editing, Funding acquisition.

## Declaration of competing interest

The authors declare that they have no known competing financial interests or personal relationships that could have appeared to influence the work reported in this paper.

## Data Availability

The authors do not have permission to share data.

## Appendix A Supplementary data

Supplementary material 1 Supplementary fugures for Effects of *Chardonnay* grape skin residue on the chemical, sensory and nutritional value of *Cabernet Sauvignon* wine. Supplementary material 1

Supplementary material 2 Non-targeted metabolites in CSW and CSWS wine samples before and after digestion. Supplementary material 2

Supplementary material 3 Original mass spectrogram of CSW wine in positive ion mode. CSW, *Cabernet Sauvignon* wine. Supplementary material 3

Supplementary material 4 Original mass spectrogram of CSW wine in negative ion mode. CSW, *Cabernet Sauvignon* wine. Supplementary material 4

Supplementary material 5 Original mass spectrogram of CSW wine in positive ion modes after in vitro simulated digestion. CSW, Cabernet Sauvignon wine. Supplementary material 5

Supplementary material 6 Original mass spectrogram of CSW wine in negative ion modes after in vitro simulated digestion. CSW, *Cabernet Sauvignon* wine. Supplementary material 6

Supplementary material 7 Original mass spectrogram of CSWS wine in negative ion modes after in vitro simulated digestion. CSWS, *Cabernet Sauvignon* wine with *Chardonnay* grape skin residue. Supplementary material 7

Supplementary material 8 Original mass spectrogram of CSWS wine in positive ion modes after in vitro simulated digestion. CSWS, *Cabernet Sauvignon* wine with *Chardonnay* grape skin residue. Supplementary material 8

Supplementary material 9 Original mass spectrogram of CSWS wine in negative ion mode. CSWS, *Cabernet Sauvignon* wine with *Chardonnay* grape skin residue. Supplementary material 9

Supplementary material 10 Original mass spectrogram of CSWS wine in positive ion mode. CSWS, *Cabernet Sauvignon* wine with *Chardonnay* grape skin residue. Supplementary material 10
