# Supplementary material for: Effects of Chardonnay grape skin residue on the chemical, sensory and nutritional value of Cabernet Sauvignon wine
Source: Food Chem X. 2025 Sep 19;31:103043. doi: 10.1016/j.fochx.2025.103043 (PMC12508854; doi:10.1016/j.fochx.2025.103043)
Supplement: Supplementary material 1 — Supplementary fugures for Effects of Chardonnay grape skin residue on the chemical, sensory and nutritional value of Cabernet Sauvignon wine. [file mmc1.docx]

**Supplementary Information for**

**Effects of *Chardonnay* grape skin residue on the chemical, sensory and nutritional value of *Cabernet Sauvignon* wine**

Xiaochun Zheng ^a, b, 1^, Jie Sheng ^b, c,^ ^1^, Yu Chen ^a, b^, Bin Wang ^b, *^, Xuewei Shi ^b, *^

1: These authors equally contributed to this study.

a: College of Enology, Northwest A&F University, Yangling 712100, Shaanxi, PR China.

b: Food College, Shihezi University, Shihezi, 832000 Xinjiang Uygur Autonomous Region, PR China.

c: College of Food Science and Technology, Huazhong Agricultural University, Wuhan, Hubei 430070, PR China.

* Corresponding authors

Mailing address: Food College, Shihezi University, Shihezi, 832000 Xinjiang Uygur Autonomous Region, PR China.

E-mail addresses: shixuewei@shzu.edu.cn; binwang0228@shzu.edu.cn.

**Supplementary captions**

**Figures: S1-S15**

**Figure S1.** Gas chromatogram of volatile substances in wine samples. A: Gas chromatogram of volatile substances in CSW; B: Gas chromatogram of volatile substances in CSWS. CSW, *Cabernet Sauvignon* wine; CSWS, CSW with *Cabernet Sauvignon* grape skin residue.

**Figure S2.** Mass spectrum of volatile substances in wine samples. A: Mass spectrum of volatile substances in CSW; B: Mass spectrum of volatile substances in CSWS. CSW, *Cabernet Sauvignon* wine; CSWS, CSW with *Cabernet Sauvignon* grape skin residue.

**Figure S3.** Mass spectrum of main volatile substances in wine samples. The mass spectrogram of the volatile substance was obtained through retrieval in the NIST14 gas chromatography-mass spectrometry library.

**Figure S4.** Metabolite chromatograms of CSW wine. A and B: CSW wine chromatograms in positive and negative ion modes, respectively. CSW, *Cabernet Sauvignon* wine.

**Figure S5.** Metabolite chromatograms of CSWS wine. A and B: CSWS wine chromatograms in positive and negative ion modes, respectively. CSWS, *Cabernet Sauvignon* wine with *Cabernet Sauvignon* grape skin residue.

**Figure S6.** Metabolite chromatograms in CSW wine after *in vitro* simulated digestion. A and B: chromatograms of CSW wine in positive and negative ion modes after *in vitro* simulated digestion., respectively. CSW, *Cabernet Sauvignon* wine.

**Figure S7.** Metabolite chromatograms in CSWS wine after *in vitro* simulated digestion. A and B: chromatograms of CSWS wine in positive and negative ion modes after *in vitro* simulated digestion., respectively. CSWS, *Cabernet Sauvignon* wine with *Cabernet Sauvignon* grape skin residue.

**Figure S8.** Influence of adding *Chardonnay* grape skin residue on the non-volatile metabolites in *Cabernet Sauvignon* wine.

**Figure S9.** Relative changes in phenolic acids in CSW and CSWS samples before and after digestion. CSW, *Cabernet Sauvignon* wine; CSWS, CSW with *Cabernet Sauvignon* grape skin residue.

**Figure S10.** Heat map of the correlations of differential metabolites (top 10). The correlation coefficient (R) between differential metabolites is between -1 and +1. An R > 0 indicates a positive correlation, which is represented by red; an R < 0 indicates a negative correlation, which is represented by blue. The larger the proportion of the colored interval, the stronger the positive/negative correlation.

**Figure S11.** Changes in differential metabolites before and after the digestion of CSWS (*Cabernet Sauvignon* wine with grape skin residue).

**Figure S12.** KEGG pathway diagram of significantly different metabolites in CSWS (*Cabernet Sauvignon* wine with grape skin residue) after *in vitro* simulated digestion experiments. A: KEGG pathway diagram of significantly different metabolites at top category; B: KEGG pathway diagram of significantly different metabolites at secondary category.

**Figure S13.** Differential abundance scores after the digestion of KEGG pathway diagram of significantly different metabolites in CSWS (*Cabernet Sauvignon* wine with grape skin residue) after *in vitro* simulated digestion experiments. A: Top catergory; B: Secondary catergory. The DA score represents the overall trend change of all metabolites in the metabolic pathway. The vertical axis represents the pathways, and different colors belong to different classifications. Each circle corresponds to one pathway, and the size of the circle indicates the number of metabolites annotated to this pathway. The color ranges from blue to red, indicating that the DA score ranges from -1 to 1. When the DA score is -1, it means that the abundance of all metabolites in this pathway decreases. When the DA score is 1, it means that the abundance of all metabolites in this pathway increases. The closer it is to 1 or -1, the more the overall expression of this pathway tends to be upregulated or downregulated.

**Figure S14.** Relationship diagram between the top 15 pathways with enrichment significance and differential metabolites in KEGG pathway diagram of significantly different metabolites in CSWS (*Cabernet Sauvignon* wine with grape skin residue) after *in vitro* simulated digestion experiments after *in vitro* simulated digestion experiments. From left to right were differential metabolites (red indicated up-regulation and blue indicated down-regulation), metabolic pathways with significant enrichment (p < 0.05), up to 15 at most, the second-level KEGG pathway categories, and the top-level KEGG pathway categories.

**Figure S15.** Bar chart of the significantly enriched SMPDB primary pathways after the digestion of KEGG pathway diagram of significantly different metabolites in CSWS (*Cabernet Sauvignon* wine with grape skin residue) after *in vitro* simulated digestion experiments. The horizontal axis represents the negative logarithmic transformation of the p-value, and the vertical axis represents the names of pathway categories. From top to bottom of the pathways, the -log10(p-value) decreases successively, that is, the p-value increases successively, and the significance decreases successively. The filling color of the bar changes from dark to light, indicating that the p-value increases and the significance decreases.


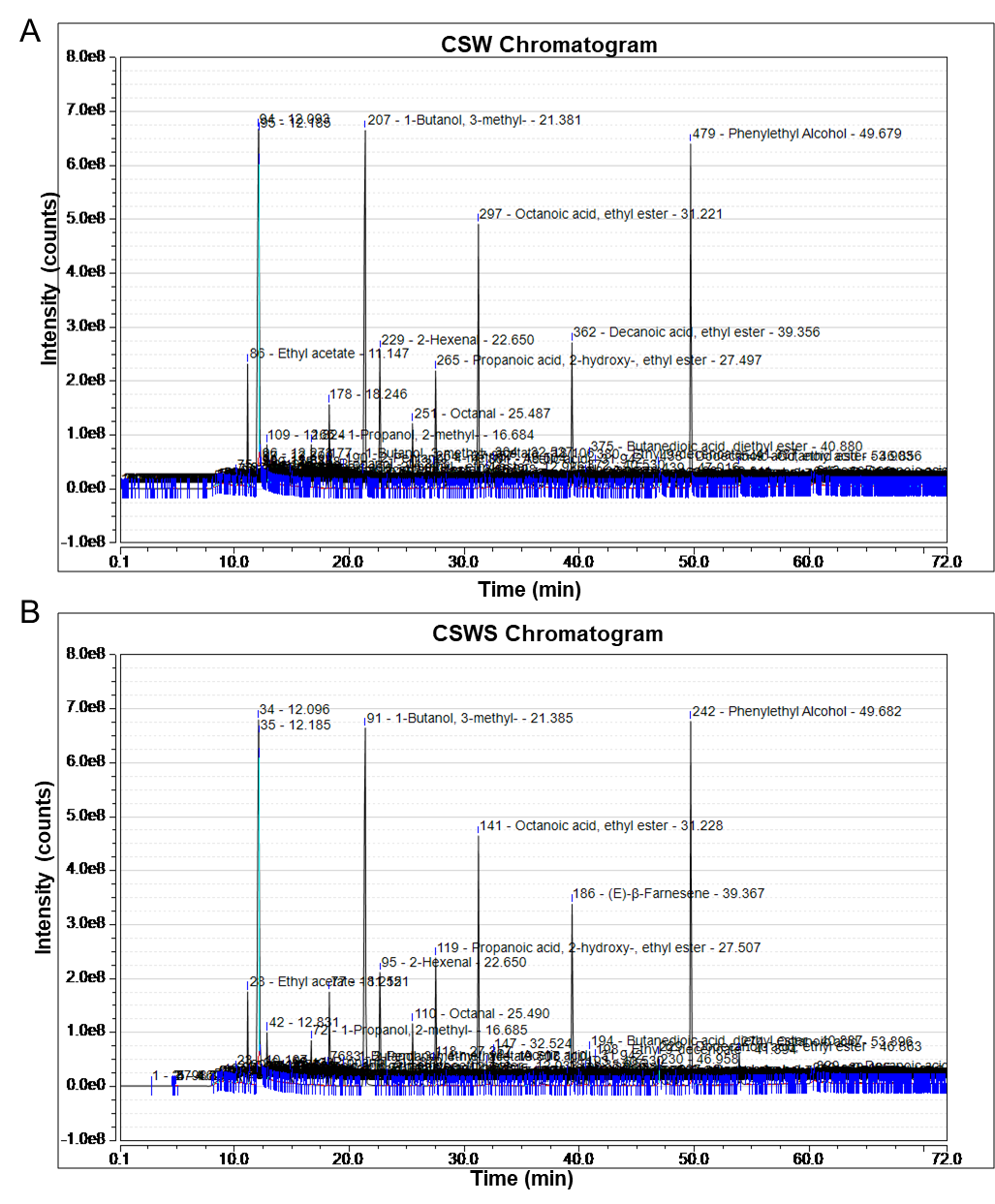


**Figure S1.** Gas chromatogram of volatile substances in wine samples. A: Gas chromatogram of volatile substances in CSW; B: Gas chromatogram of volatile substances in CSWS. CSW, *Cabernet Sauvignon* wine; CSWS, CSW with *Cabernet Sauvignon* grape skin residue.


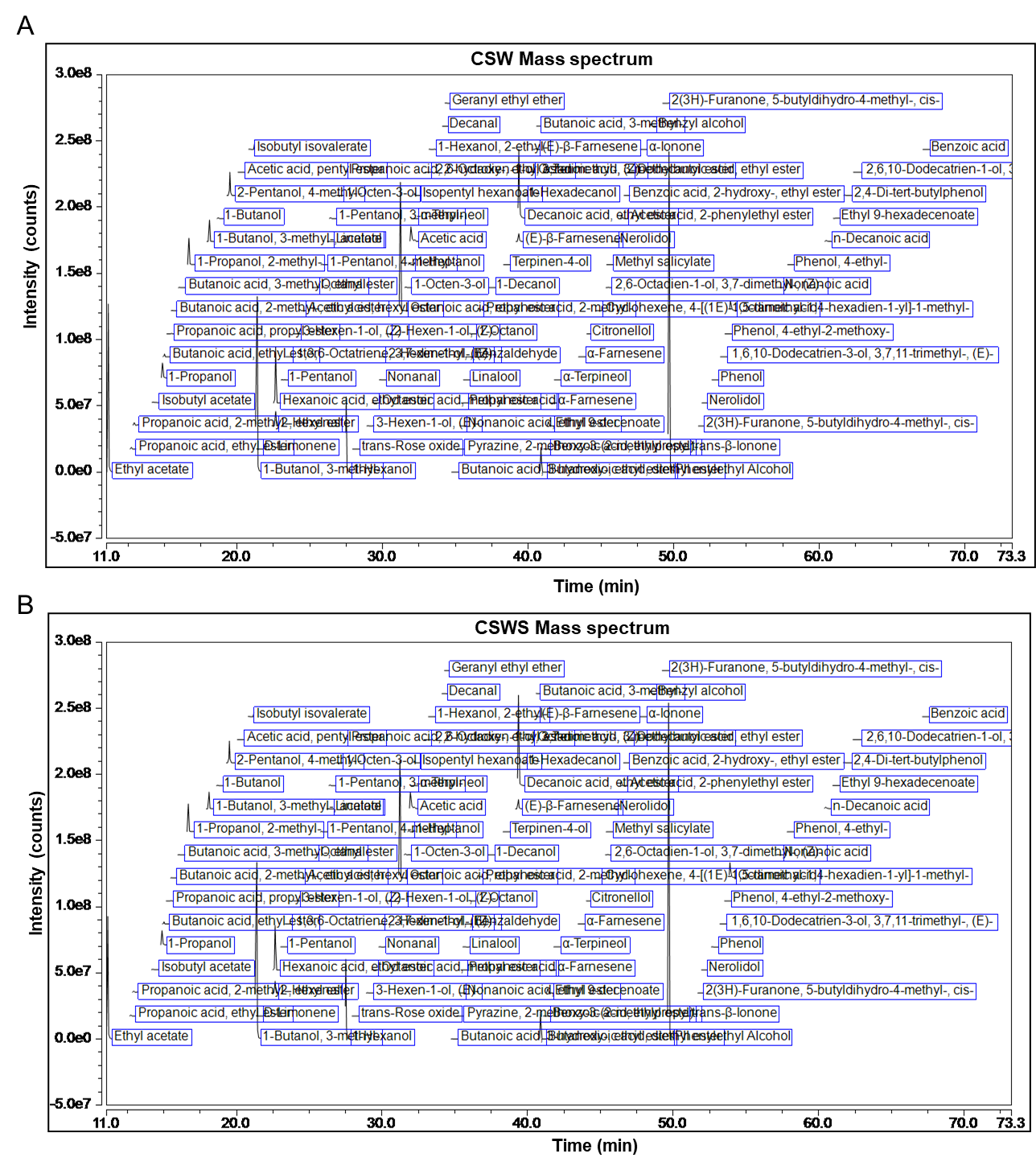


**Figure S2.** Mass spectrum of volatile substances in wine samples. A: Mass spectrum of volatile substances in CSW; B: Mass spectrum of volatile substances in CSWS. CSW, *Cabernet Sauvignon* wine; CSWS, CSW with *Cabernet Sauvignon* grape skin residue.

| 1-Propanol   |
| --- |
| 2-Methyl-1-propanol   |
| 1-Butanol   |
| 4-Methyl-2-pentanol   |
| 3-Methyl-1-butanol   |
| (Z)-3-Hexen-1-ol   |
| 4-Methyl-1-pentanol   |
| 3-Methyl-1-pentanol   |
| 1-Octen-3-ol   |
| 1-Hexanol   |
| (E)-3-Hexen-1-ol   |
| (Z)-2-Hexen-1-ol   |
| 1-Heptanol   |
| α-Terpineol   |
| Linalool   |
| Nerol   |
| 1-Decanol   |
| 1-Hexadecanol   |
| Benzyl alcohol   |
| Phenylethyl alcohol   |
| (Z,E)-farnesol |
|  |
| Ethyl acetate   |
| Ethyl butanoate   |
| Isobutyl isovalerate   |
| Ethyl hexanoate   |
| Ethyl caprylate   |
| Ethyl 3-hydroxybutyrate   |
| Ethyl caprate   |
| Diethyl succinate   |
| Ethyl benzoate |
|  |
| Phenethyl acetate   |
| Isoamyl acetate   |
| Ethyl 9-decenoate |
|  |
| Propanoic acid   |
| 2-Methyl-propanoic acid   |
| 3-Methyl-butanoic acid   |
| Octanoic acid   |
| n-Decanoic acid   |
| Benzoic acid |
|  |
| Phenol   |
| 4-Ethyl-2-methoxyphenol   |
| 4-Ethyl-phenol   |
| 2,4-Di-t-butylphenol   |
|  |
| (E)-β-Farnesene |
|  |
| 2-Hexenal |
|  |
| (Z)-oak lactone   |

**Figure S3.** Mass spectrum of main volatile substances in wine samples. The mass spectrogram of the volatile substance was obtained through retrieval in the NIST17.0 gas chromatography-mass spectrometry library.


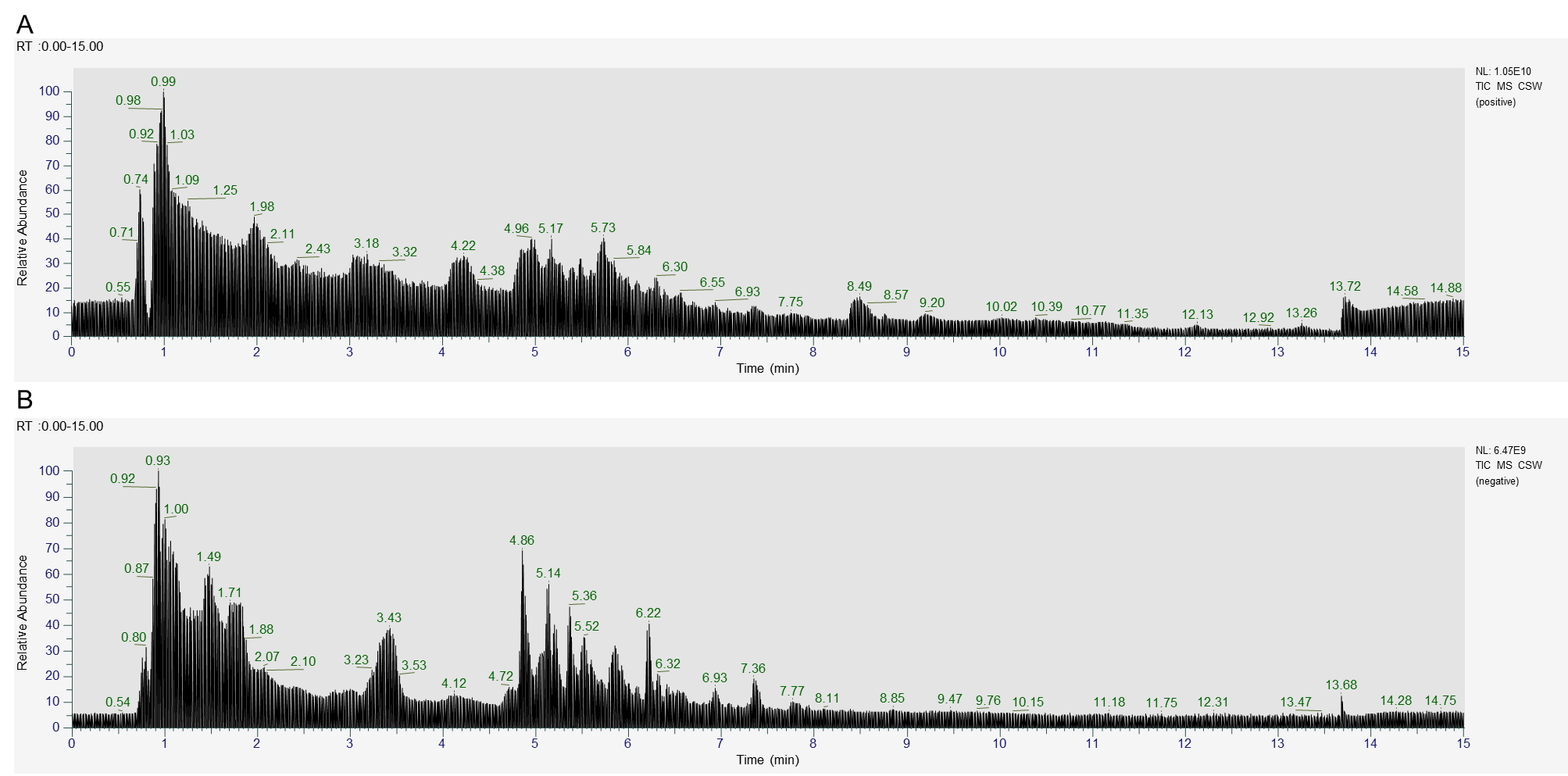


**Figure S4.** Metabolite chromatograms of CSW wine. A and B: CSW wine chromatograms in positive and negative ion modes, respectively. CSW, *Cabernet Sauvignon* wine.


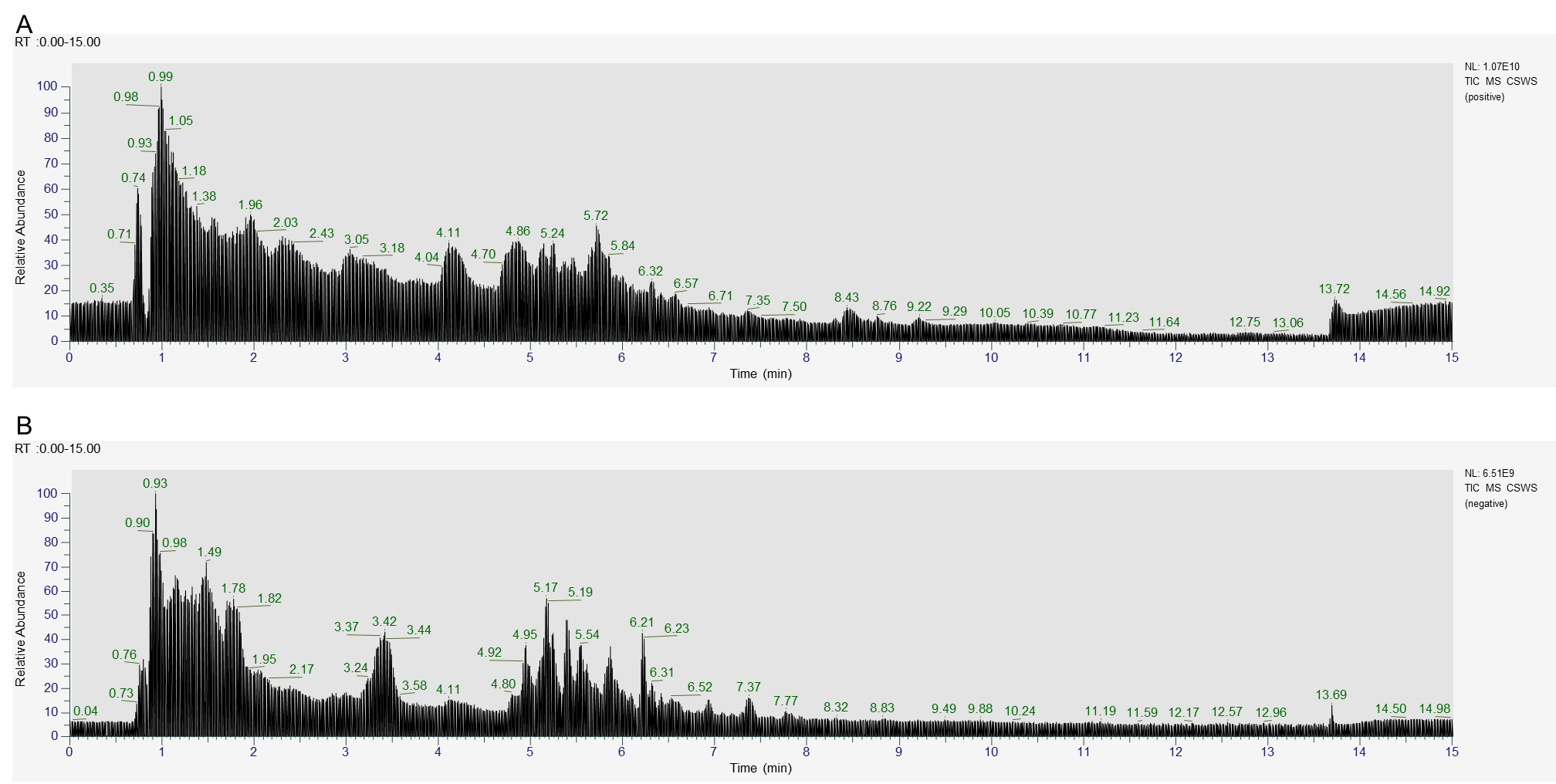


**Figure S5.** Metabolite chromatograms of CSWS wine. A and B: CSWS wine chromatograms in positive and negative ion modes, respectively. CSWS, *Cabernet Sauvignon* wine with *Cabernet Sauvignon* grape skin residue.


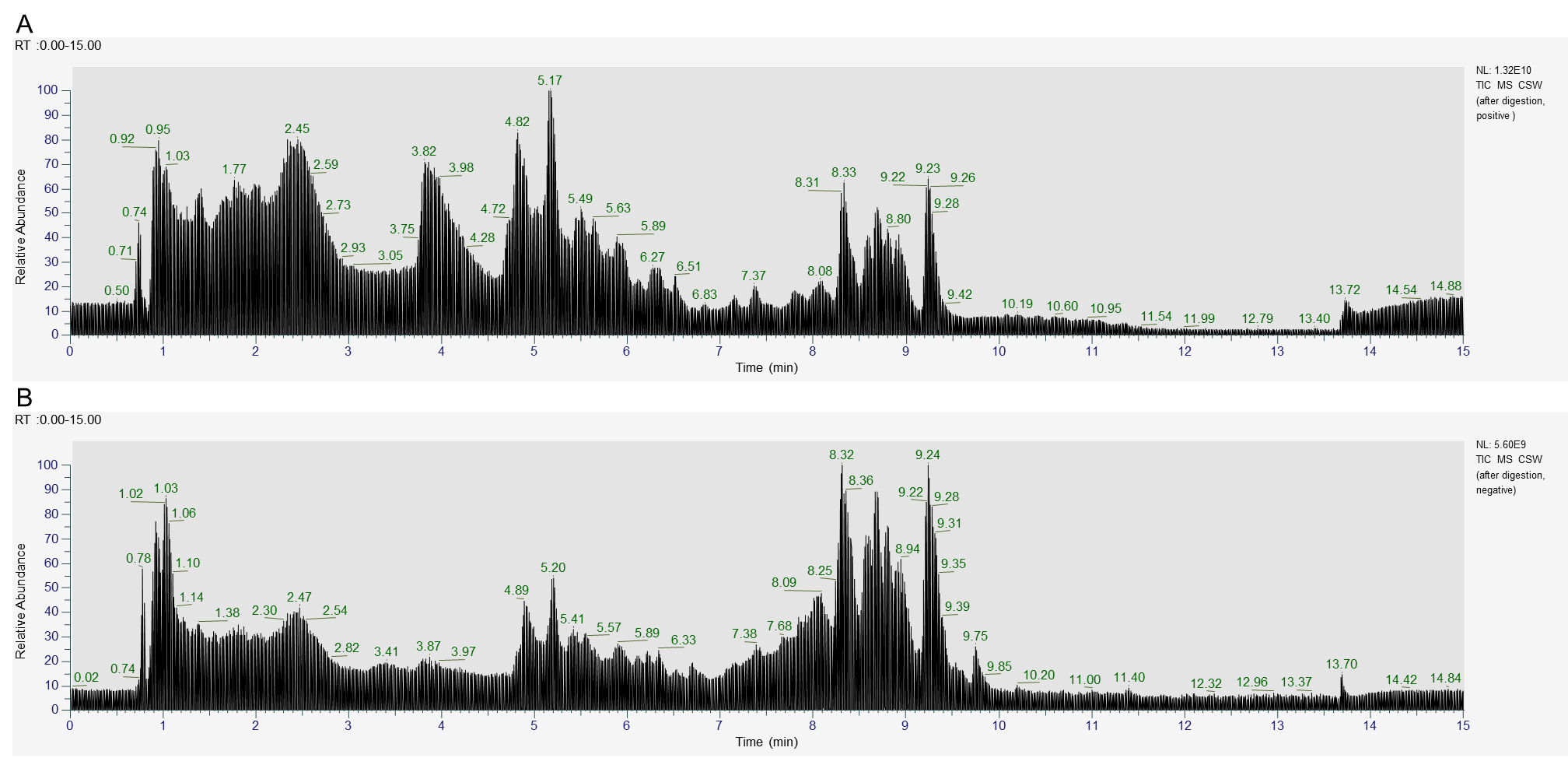


**Figure S6.** Metabolite chromatograms in CSW wine after *in vitro* simulated digestion. A and B: chromatograms of CSW wine in positive and negative ion modes after *in vitro* simulated digestion., respectively. CSW, *Cabernet Sauvignon* wine.


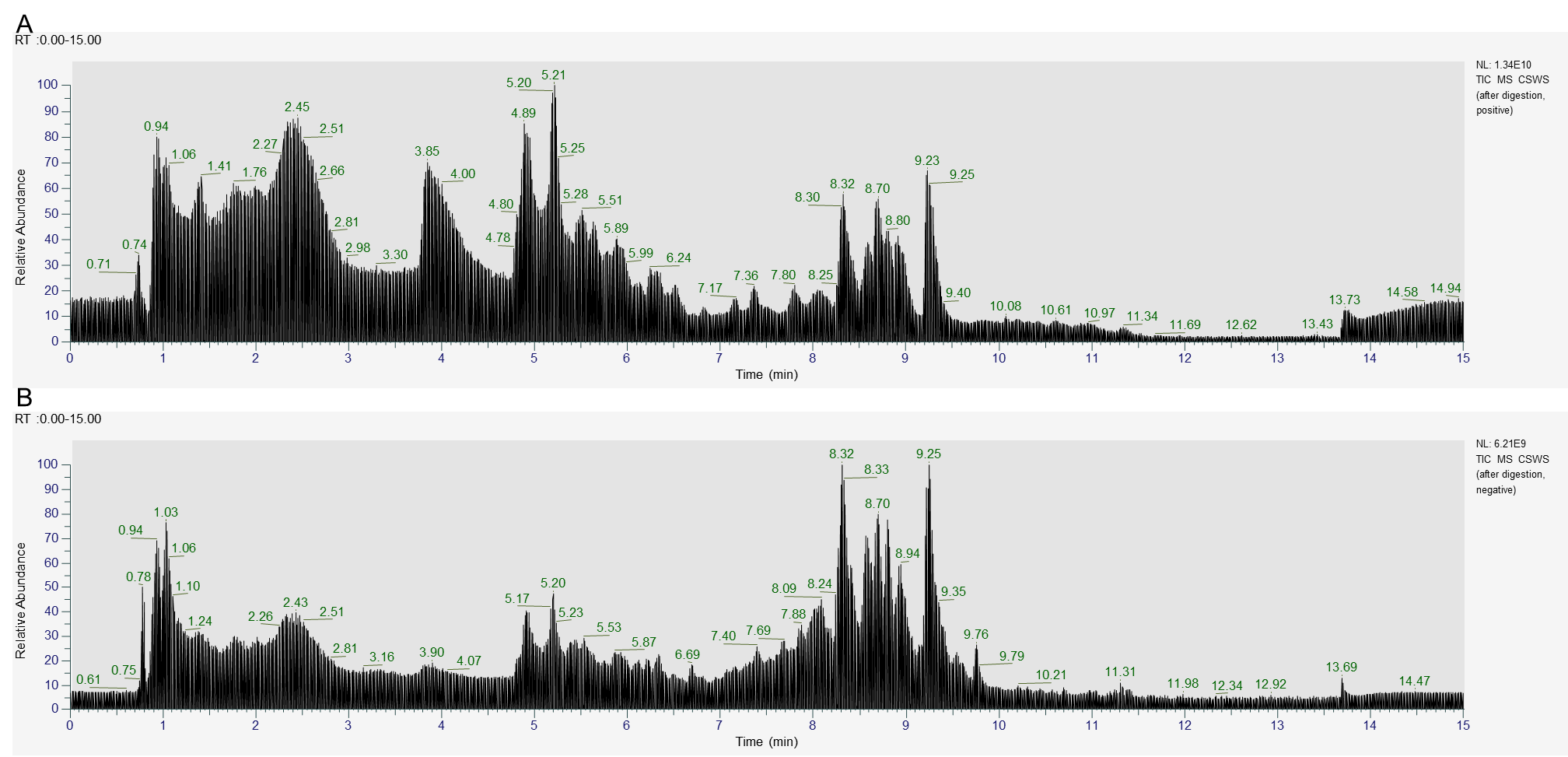


**Figure S7.** Metabolite chromatograms in CSWS wine after *in vitro* simulated digestion. A and B: chromatograms of CSWS wine in positive and negative ion modes after *in vitro* simulated digestion., respectively. CSWS, *Cabernet Sauvignon* wine with *Cabernet Sauvignon* grape skin residue.


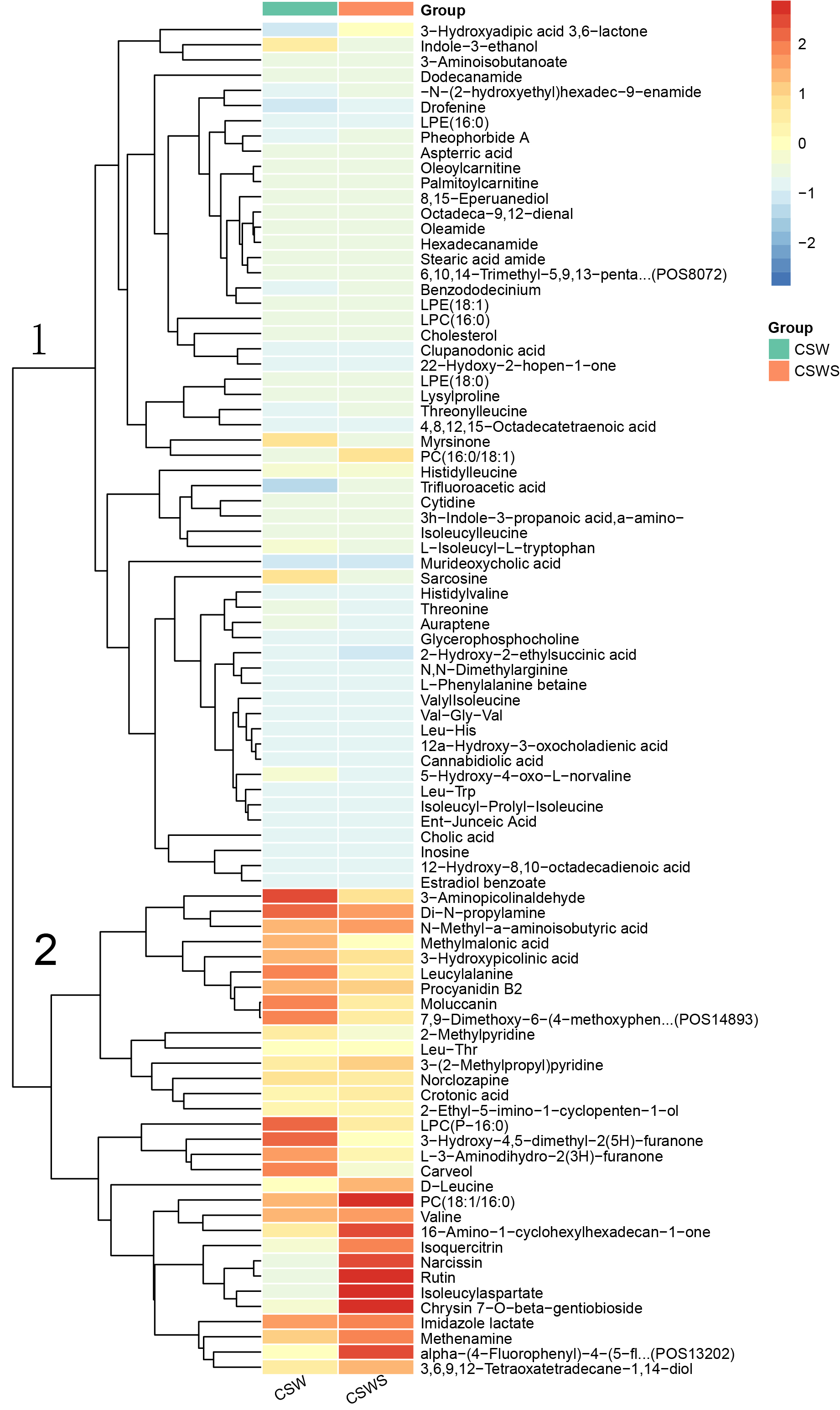


**Figure S8.** Influence of adding *Chardonnay* grape skin residue on the non-volatile metabolites in *Cabernet Sauvignon* wine.


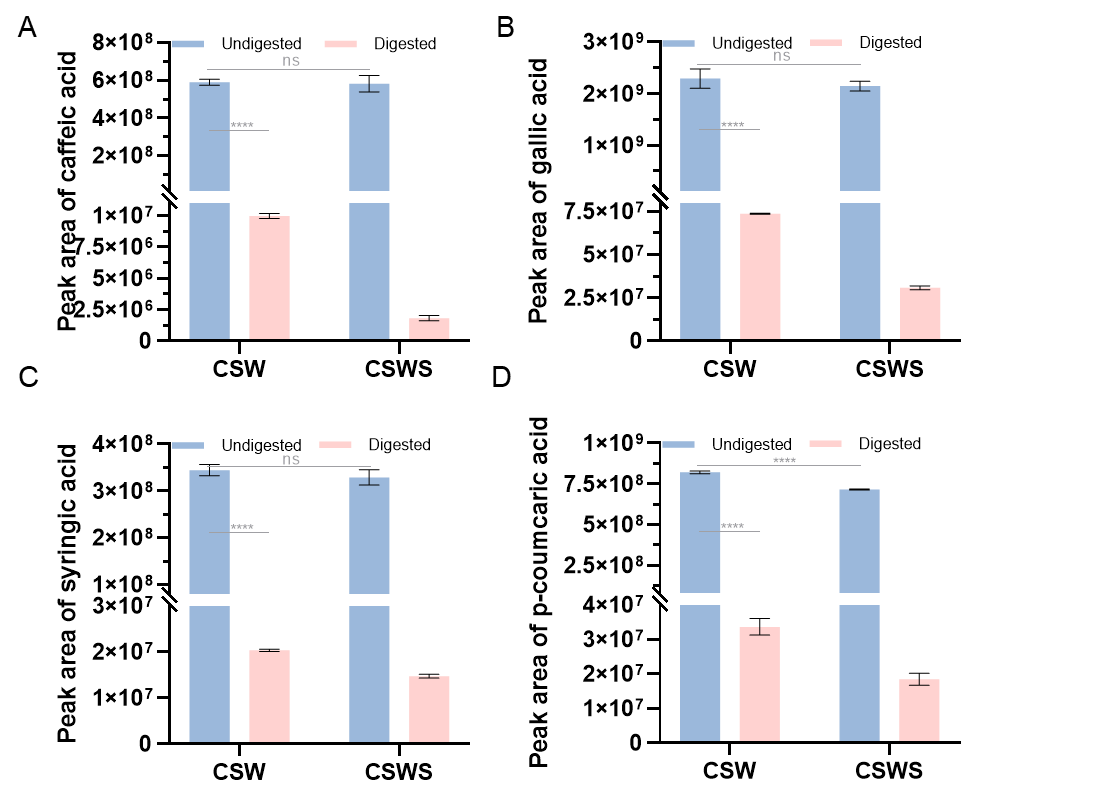


**Figure S9.** Relative changes in phenolic acids in CSW and CSWS samples before and after digestion. CSW, *Cabernet Sauvignon* wine; CSWS, CSW with *Cabernet Sauvignon* grape skin residue. The results were statistically significant (p < 0.05). All data were presented as means and standard derivations.


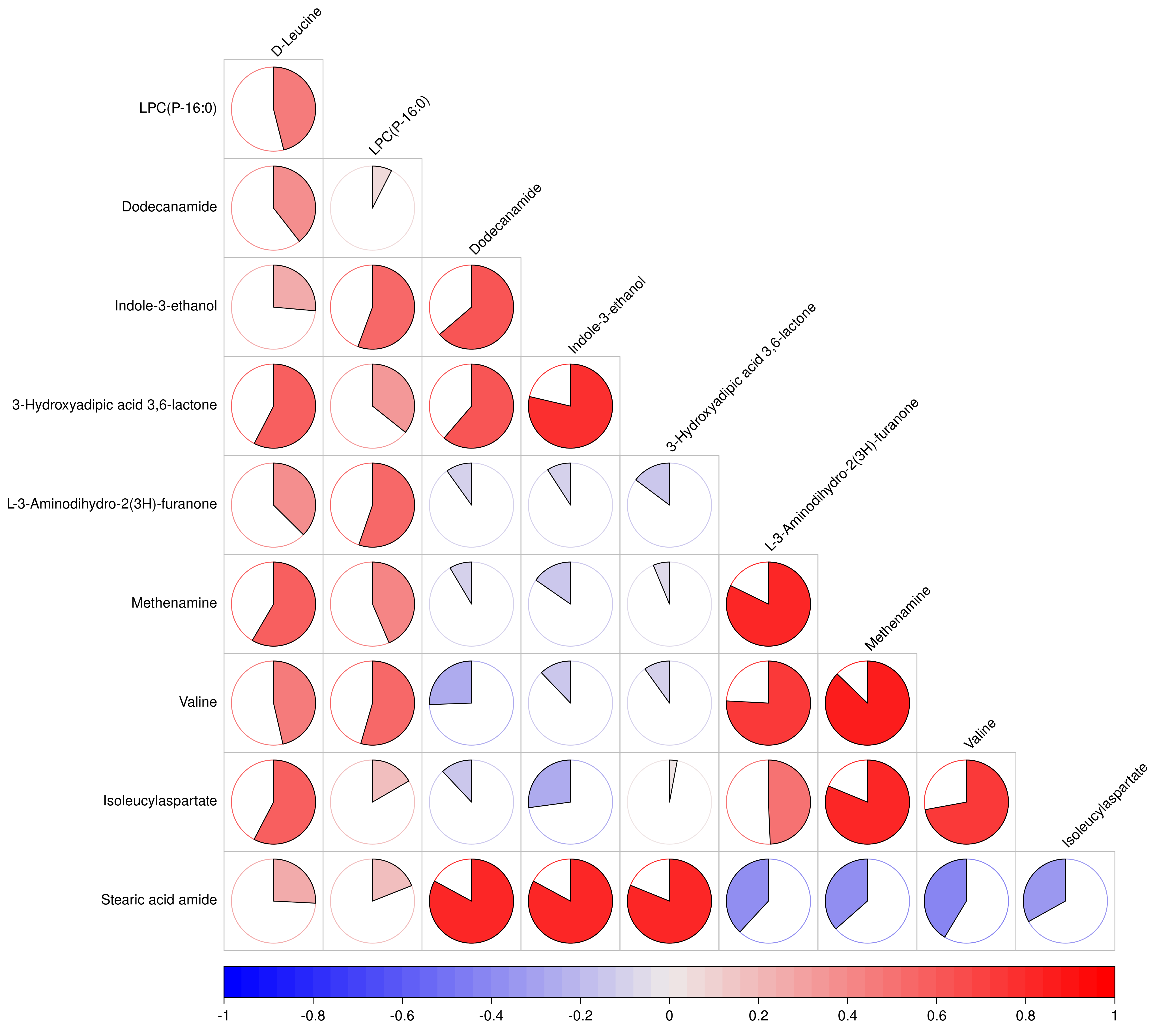


**Figure S10.** Heat map of the correlations of differential metabolites (top 10). The correlation coefficient (R) between differential metabolites is between -1 and +1. An R > 0 indicates a positive correlation, which is represented by red; an R < 0 indicates a negative correlation, which is represented by blue. The larger the proportion of the colored interval, the stronger the positive/negative correlation.


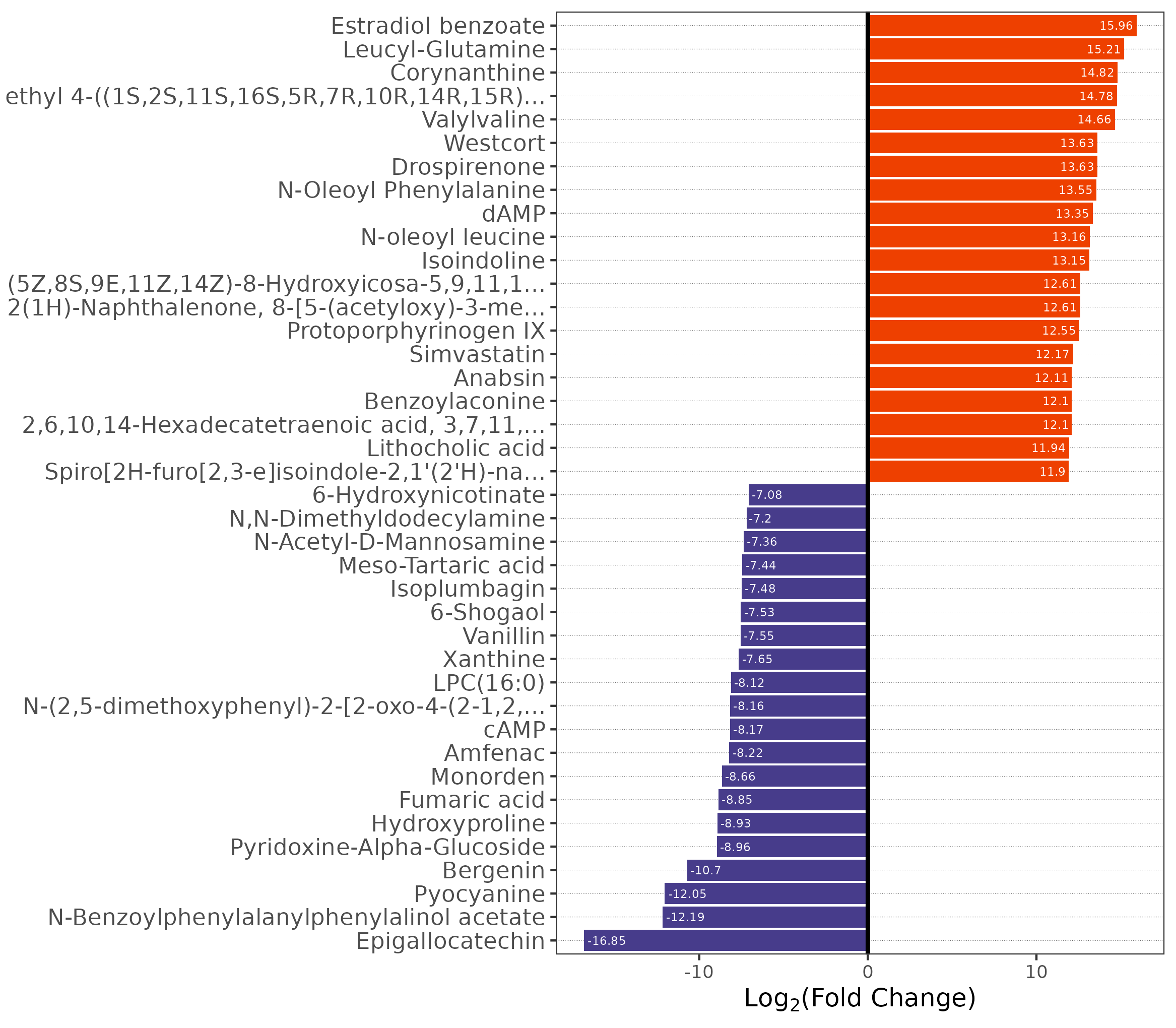


**Figure S11.** Changes in differential metabolites before and after the digestion of CSWS (*Cabernet Sauvignon* wine with grape skin residue).


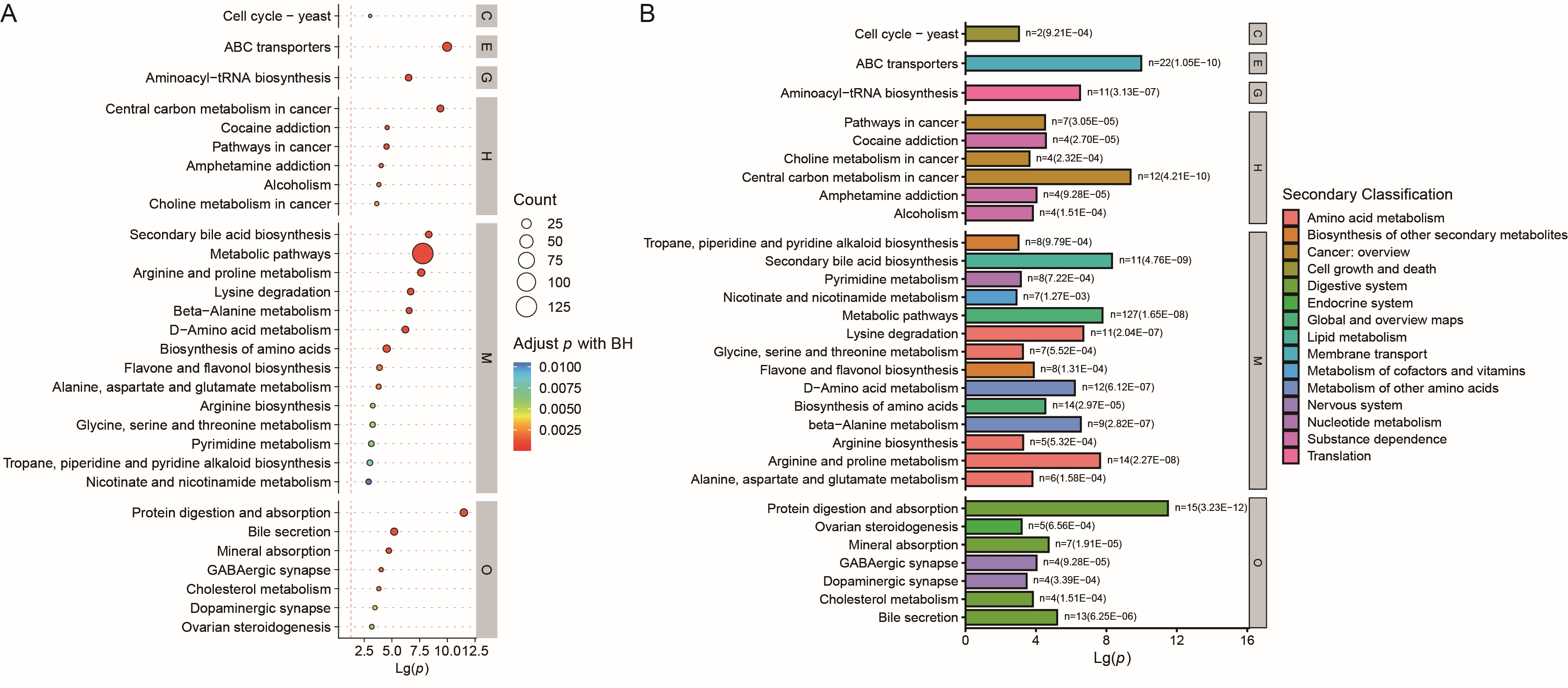


**Figure S12.** KEGG pathway diagram of significantly different metabolites in CSWS (*Cabernet Sauvignon* wine with grape skin residue) after *in vitro* simulated digestion experiments. A: KEGG pathway diagram of significantly different metabolites at top category; B: KEGG pathway diagram of significantly different metabolites at secondary category.


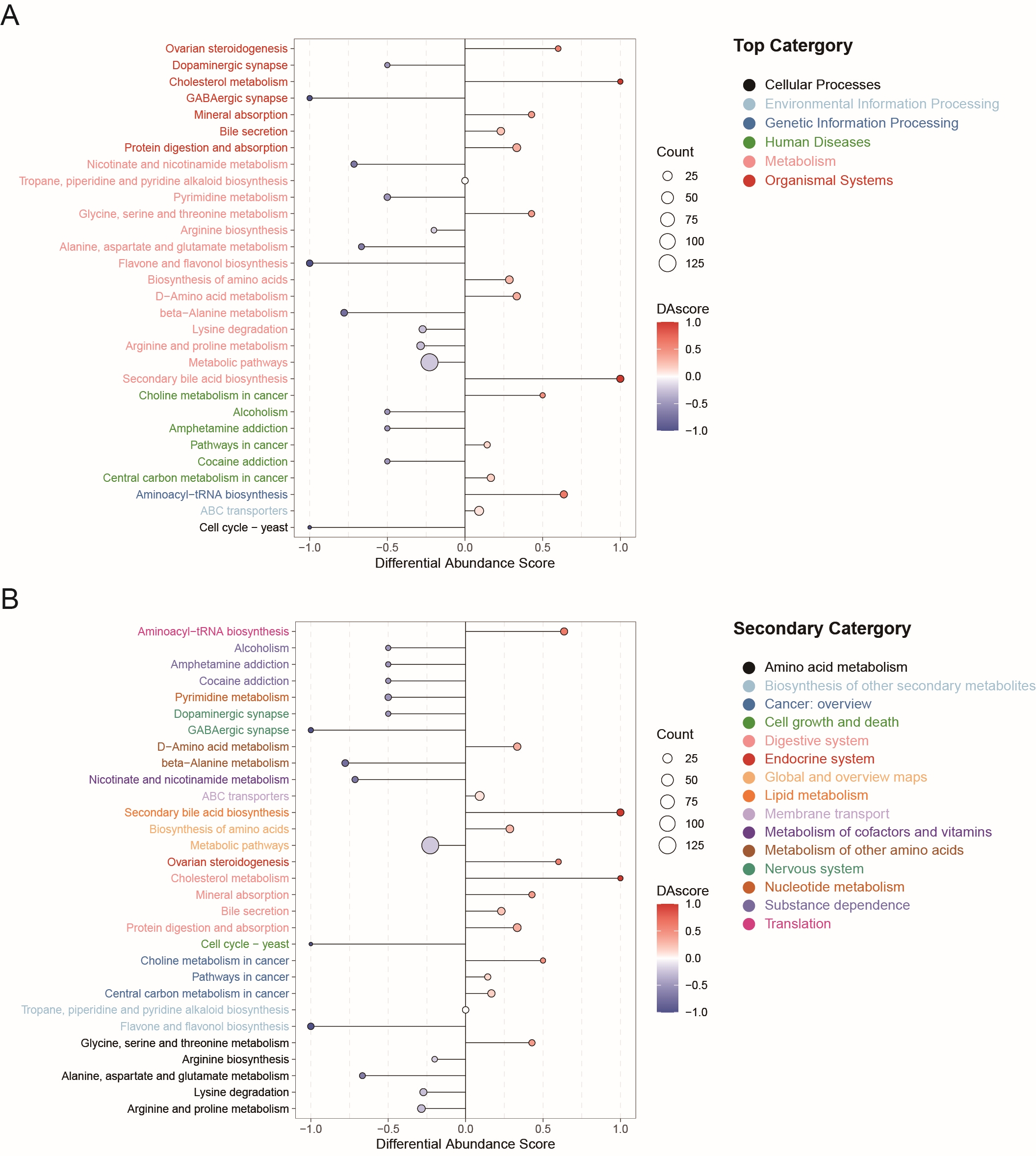


**Figure S13.** Differential abundance scores after the digestion of KEGG pathway diagram of significantly different metabolites in CSWS (*Cabernet Sauvignon* wine with grape skin residue) after *in vitro* simulated digestion experiments. A: Top catergory; B: Secondary catergory. The DA score represents the overall trend change of all metabolites in the metabolic pathway. The vertical axis represents the pathways, and different colors belong to different classifications. Each circle corresponds to one pathway, and the size of the circle indicates the number of metabolites annotated to this pathway. The color ranges from blue to red, indicating that the DA score ranges from -1 to 1. When the DA score is -1, it means that the abundance of all metabolites in this pathway decreases. When the DA score is 1, it means that the abundance of all metabolites in this pathway increases. The closer it is to 1 or -1, the more the overall expression of this pathway tends to be upregulated or downregulated.


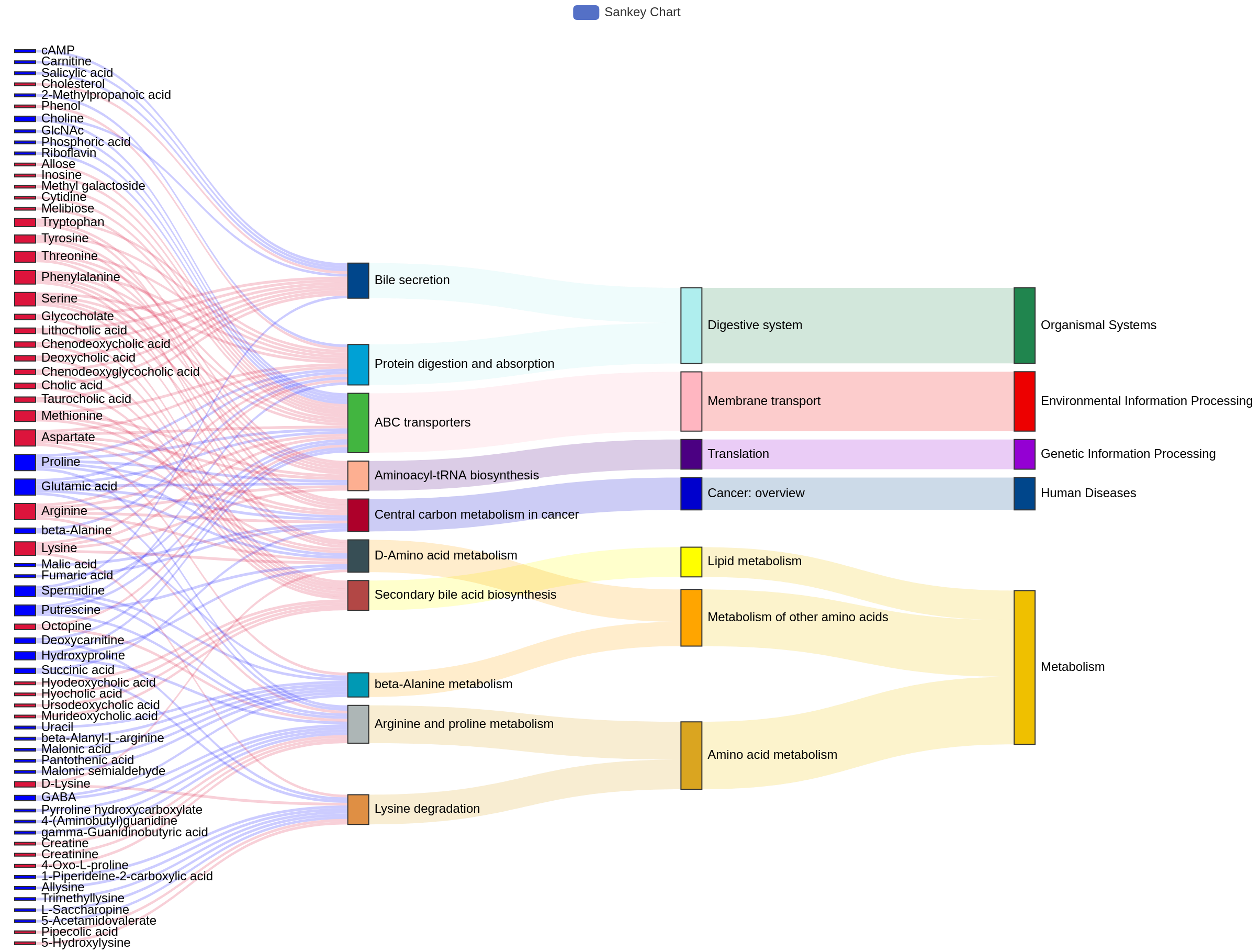


**Figure S14.** Relationship diagram between the top 15 pathways with enrichment significance and differential metabolites in KEGG pathway diagram of significantly different metabolites in CSWS (*Cabernet Sauvignon* wine with grape skin residue) after *in vitro* simulated digestion experiments after *in vitro* simulated digestion experiments. From left to right were differential metabolites (red indicated up-regulation and blue indicated down-regulation), metabolic pathways with significant enrichment (p < 0.05), up to 15 at most, the second-level KEGG pathway categories, and the top-level KEGG pathway categories.


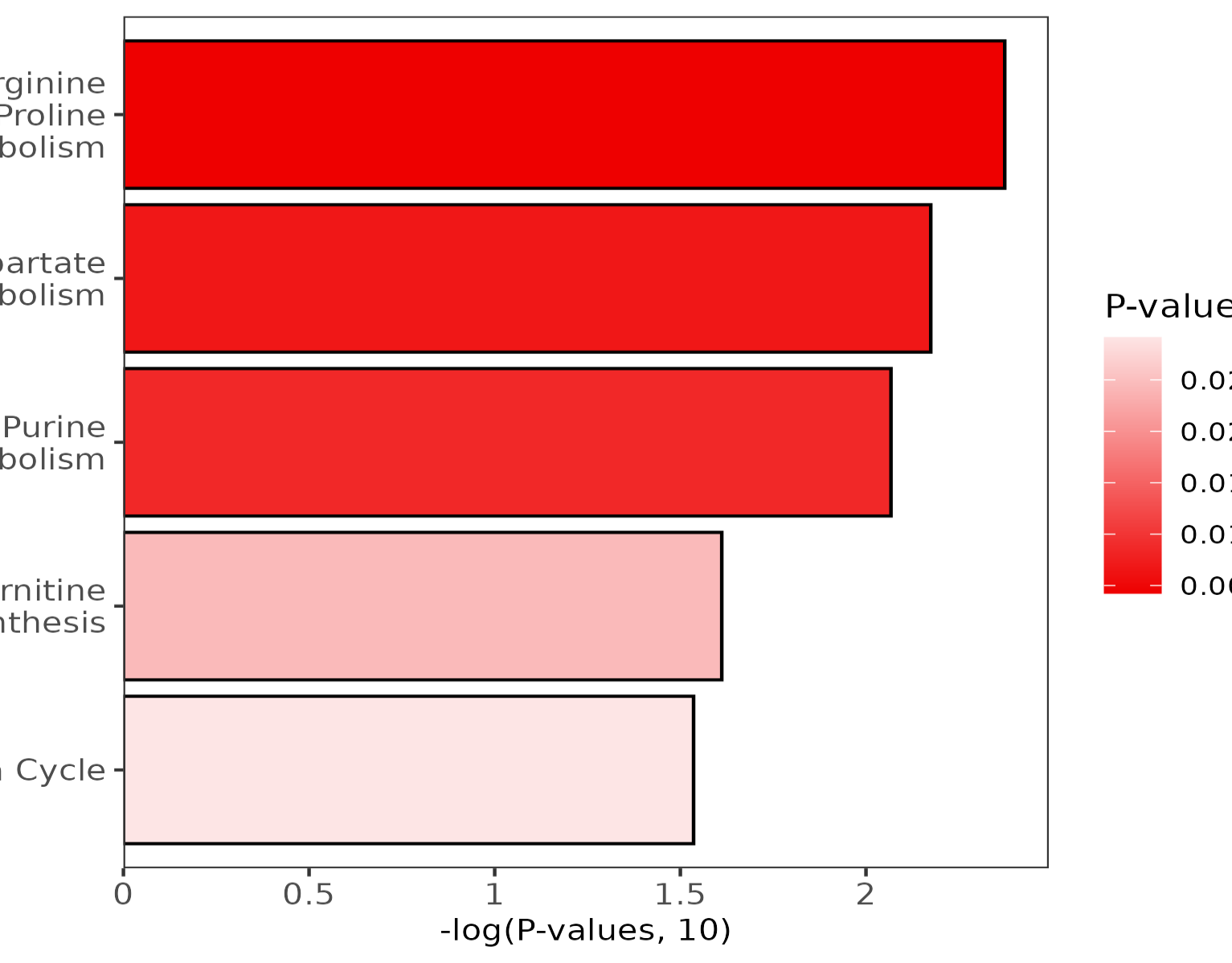


**Figure S15.** Bar chart of the significantly enriched SMPDB primary pathways after the digestion of KEGG pathway diagram of significantly different metabolites in CSWS (*Cabernet Sauvignon* wine with grape skin residue) after *in vitro* simulated digestion experiments. The horizontal axis represents the negative logarithmic transformation of the p-value, and the vertical axis represents the names of pathway categories. From top to bottom of the pathways, the -log10(p-value) decreases successively, that is, the p-value increases successively, and the significance decreases successively. The filling color of the bar changes from dark to light, indicating that the p-value increases and the significance decreases.
